# Lipid Interactions of a Ciliary Membrane TRP Channel: Simulation and Structural Studies of Polycystin-2

**DOI:** 10.1016/j.str.2019.11.005

**Published:** 2020-02-04

**Authors:** Qinrui Wang, Robin A. Corey, George Hedger, Prafulla Aryal, Mariana Grieben, Chady Nasrallah, Agnese Baronina, Ashley C.W. Pike, Jiye Shi, Elisabeth P. Carpenter, Mark S.P. Sansom

**Affiliations:** 1 Department of Biochemistry, University of Oxford, South Parks Road, Oxford OX1 3QU, UK; 2 Structural Genomics Consortium, University of Oxford, Old Road Campus Research Building, Roosevelt Drive, Oxford OX3 7DQ, UK; 3 UCB Pharma, 208 Bath Road, Slough SL1 3WE, UK

**Keywords:** TRP channel, lipids, cryoelectron microscopy, molecular dynamics, cholesterol, phosphatidylinositol bisphosphate, polycystin-2

## Abstract

Polycystin-2 (PC2) is a transient receptor potential (TRP) channel present in ciliary membranes of the kidney. PC2 shares a transmembrane fold with other TRP channels, in addition to an extracellular domain found in TRPP and TRPML channels. Using molecular dynamics (MD) simulations and cryoelectron microscopy we identify and characterize PIP_2_ and cholesterol interactions with PC2. PC2 is revealed to have a PIP binding site close to the equivalent vanilloid/lipid binding site in the TRPV1 channel. A 3.0-Å structure reveals a binding site for cholesterol on PC2. Cholesterol interactions with the channel at this site are characterized by MD simulations. The two classes of lipid binding sites are compared with sites observed in other TRPs and in Kv channels. These findings suggest PC2, in common with other ion channels, may be modulated by both PIPs and cholesterol, and position PC2 within an emerging model of the roles of lipids in the regulation and organization of ciliary membranes.

## Highlights

• Lipid interactions of PC2 channels have been explored by MD simulation and cryo-EM
• PIP_2_ binds to a site corresponding to the vanilloid/lipid binding site of TRPV1
• Cholesterol binds between the S3 and S4 helices and S6 of the adjacent subunit
• PC2, in common with other channels, may be modulated by PIPs and cholesterol

## Graphical Abstract

**Figure:**
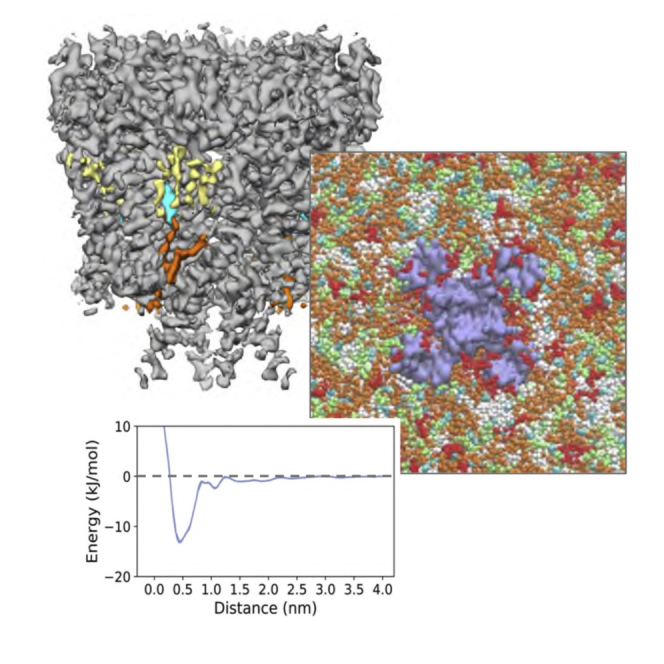

## Teaser

Wang et al. use molecular dynamics simulations and cryoelectron microscopy to explore interactions of the PC2 channel with lipids. Phosphatidylinositol phosphates (PIPs) bind to a site corresponding to the vanilloid/lipid binding site of TRPV1, whereas cholesterol binds to a different site. This suggests PC2 may be modulated by PIPs and cholesterol.

## Introduction

Ion channels are of considerable importance in numerous aspects of cell physiology (Hille, 2001), and mutations in channels cause many human diseases (Bagal et al., 2013). The transient receptor potential (TRP) superfamily of non-selective cation channels is a major class of ion channels found in all eukaryotes. They are involved in many aspects of cellular function, including thermosensation, osmotic pressure regulation, mechanosensation, and detection of noxious substances (Vetter and Lewis, 2011). TRP channels are activated and inhibited by a range of mechanisms, in response to thermal or chemical stimuli and/or mechanical forces (Venkatachalam and Montell, 2007). TRP channel mutations have been implicated in a number of different diseases (Nilius and Owsianik, 2010) and consequently are of interest as potential drug targets (Moran, 2018). Several TRP channels are modulated by membrane lipids (e.g., Fine et al., 2018, Wilkes et al., 2017, Yin et al., 2019), suggesting the possibility of lipid-based therapies (Ciardo and Ferrer-Montiel, 2017).

Mammalian TRP channels can be divided into five families, the TRPC (classical or canonical), TRPV (Vanilloid), TRPM (Melastatin), TRPP (Polycystin), TRPML (Mucolipin), and TRPA (Ankyrin) subfamilies (Rohacs, 2009). Structurally, all TRP channels have a tetrameric architecture assembled from identical or similar subunits. Each of the four subunits is composed of six transmembrane (TM) helices (S1–S6) with a pore loop region between S5 and S6 (Cao et al., 2013, Gao et al., 2016, Grieben et al., 2017, Huynh et al., 2016, Jin et al., 2017, Liao et al., 2013, Paulsen et al., 2015, Saotome et al., 2016, Shen et al., 2016, Su et al., 2018b, Wilkes et al., 2017, Zubcevic et al., 2016). TM helices S1 to S4 form a voltage sensor-like domain (VSLD), which is packed against the pore domain (S5-Pore-S6) of the adjacent chain, as is also seen in Kv channels, where structural data reveal that lipids mediate interactions of the voltage sensor domain with the pore (Long et al., 2007).

As a member of the TRPP subfamily, the polycystin-2 (PC2, also known as PKD2 or TRPP1) homo-tetramer has the same fold as other TRP channels. An extracellular domain (referred to as the TOP domain) is formed from a 218-residue insertion between S1 and S2 plus a 20-residue insertion between S3 and S4 of the VSLD (Grieben et al., 2017, Shen et al., 2016, Wilkes et al., 2017). These structures have a remarkably similar fold, despite the fact that they were solved in a range of environments (detergent, nanodiscs, and amphipols), with both truncated and full-length protein, and it is interesting that even with full-length protein, the structures observed in the maps were of very similar length. Structures of the closely related TRPP2L1 protein (Hulse et al., 2018, Su et al., 2018b), and also of the PC1/PC2 1:3 hetero-tetrameric complex (Su et al., 2018a) have also recently been determined.

Mutations in PC2 are responsible for approximately 15% of autosomal dominant polycystic kidney disease (ADPKD), which is one of the most prevalent genetic disorders in humans, affecting 4 to 6 million people worldwide (Wilson, 2004). Most other cases of ADPKD (70%) are caused by mutations in PC1, which forms a 1:3 complex with PC2 by replacing one PC2 subunit in the channel region (Su et al., 2018a). ADPKD is characterized by formation and progressive enlargement of fluid-filled renal cysts in both kidneys, which ultimately causes kidney failure (Pavel et al., 2016). Cysts or diverticula also frequently develop in intestines, liver, and pancreas (Wilson, 2004). In addition, ADPKD is associated with increased risk of cardiovascular dysfunction including aortic aneurysms, hypertension, and heart-valve defects (Boucher and Sandford, 2004). Genetic data analysis has shown that there are at least 278 mutations in PC2 associated with ADPKD (see http://pkdb.pkdcure.org) (Gout et al., 2007). However, the underlying mechanisms by which these mutations lead to ADPKD are still poorly understood.

PC2 is widely distributed with relatively high expression in tubules within kidney cells (Chauvet et al., 2002), and was originally proposed to contribute to the transduction of extracellular mechanical stimuli caused by bending of the cilia into intracellular Ca^2+^ signals in the primary cilia of kidney epithelium (Nauli et al., 2003). In addition to its role in primary cilia, it has been proposed that PC2 could function as an intracellular Ca^2+^ release channel in the endoplasmic reticulum membrane (Koulen et al., 2002). However, more recent studies have suggested that Ca^2+^ signaling may not be involved in ciliary mechanosensation (Delling et al., 2016), and that ciliary PC2 is a non-selective Na^+^,K^+^ channel, with low levels of Ca^2+^ currents, rather than a Ca^2+^ channel (Liu et al., 2018). PC2 and PC2-like channels have been identified across a range of organisms, from yeast to humans, highlighting the wider importance of this class of proteins (Shen et al., 2016). PC2 is also important during development, being required for correct organization of the organs in left-right patterning, due to a requirement for PC2 for coordinated movement of motile cilia in a region of the embryo known as the node (Busch et al., 2017). The membranes of primary cilia have a complex organization, including differences in membrane lipid composition between the base and the main body of the cilium (Garcia et al., 2018). Deletion of PC2 in mice leads to the formation of cilia that are 5-fold longer than normal cilia. Given the involvement of membrane lipid composition in regulation and signaling in primary cilia (Tsuji et al., 2019) and observations of lipid-like density in cryoelectron microscopy (cryo-EM) structures of PC2 (Wilkes et al., 2017), therefore, it is important to establish how PC2 interacts with specific lipids in its membrane environment.

Lipids, both phospholipids and cholesterol, have been extensively studied as modulators of ion channels and membrane receptor proteins (Duncan et al., 2019). A number of anionic phospholipids, in particular phosphatidylinositol 4,5-bisphosphate (PIP_2_), have been shown to interact with and regulate ion channels (Basak et al., 2017, Hansen, 2015). G protein-coupled receptors (GPCRs) have also been shown to be allosterically regulated by anionic lipids (Dawaliby et al., 2016), and mass spectrometry and molecular dynamics (MD) simulation studies have revealed functionally important interactions of PIP_2_ with a number of class A GPCRs (Yen et al., 2018). An intensively studied example of PIP_2_ regulation of a channel is provided by the inward rectifying potassium (Kir) channels, which are activated by PIP_2_ (Hansen, 2015, Shyng et al., 2000). PIP_2_ regulation has been suggested for almost all subfamilies of TRP channels, despite their possibly diverse activation mechanisms in response to different stimuli (Brauchi et al., 2007, Rohacs, 2007, Rohacs, 2009, Steinberg et al., 2014). Positive regulation by PIP_2_ has been indicated in at least five ion channels of the TRPM subfamily (Daniels et al., 2009, Liu and Liman, 2003, Runnels et al., 2002, Toth et al., 2015, Zhang et al., 2005). For TRPV channels, activation by PIP_2_ in excised patches has been reported for TRPV1 (Klein et al., 2008), TRPV5 (Lee et al., 2005), and TRPV6 (Thyagarajan et al., 2008). Indirect inhibition of TRPV1 by PIP_2_ in intact cells has also been reported (Thyagarajan et al., 2008). Recent cryo-EM structures have revealed PIP_2_ binding sites on TRPV5 (Hughes et al., 2018b), TRPML1 (Fine et al., 2018), and TRPM8 (Yin et al., 2019). Given the importance of PIP_2_ distribution and dynamics in the membranes of primary cilia (Nakatsu, 2015), we decided to explore possible interactions of this anionic lipid with PC2. Cholesterol has also been shown to play a key role in signaling in ciliary membranes (Luchetti et al., 2016) and in their organization (Garcia et al., 2018). Because cholesterol is known to interact with many ion channels (Levitan et al., 2014, Morales-Lazaro and Rosenbaum, 2017) and receptors (Duncan et al., 2019, Lee, 2018, Oates and Watts, 2011) we decided to also explore its possible interactions with PC2.

MD simulations provide an important tool for defining lipid interactions with membrane proteins (Corradi et al., 2018, Hedger and Sansom, 2016). For example, they have been used to predict PIP_2_ binding sites on Kir channels (Schmidt et al., 2013, Stansfeld et al., 2009) and GPCRs (Yen et al., 2018), and to explore allosteric modulation of GPCRs by cholesterol (Manna et al., 2016). Given the growing number of structures of ion channels, binding affinities and specificity of interactions with lipids can be studied *in silico* via MD simulations (Domański et al., 2017, Hedger et al., 2016, Hedger et al., 2019) to provide an indication of possible mechanisms of activation and allosteric modulation of channels by lipids.

Here we use a combination of MD simulations and cryo-EM to identify and characterize PIP_2_ and cholesterol interactions with PC2. Simulations predict a phospholipid binding site corresponding to lipid-like density observed in cryo-EM maps, and free energy calculations suggest that this binding site is selective for PIP molecules over other phospholipids. The proposed PIP_2_ binding site is close to the equivalent vanilloid/lipid binding site in the TRPV1 channel (Gao et al., 2016). We also identify a binding site for cholesterol in PC2 located between the VSLD and pore domain. This binding site may be compared with cholesterol sites observed in other TRP channels and in Kv channels. Together, these results suggest that PC2, in common with other ion channels, may be modulated by both PIPs and cholesterol, and thus locate PC2 within an emerging model of the complex roles of lipids in the regulation and organization of ciliary membranes (Weiss et al., 2019).

## Results and Discussion

### A Possible Phospholipid Interaction Site Suggested by Simulations

**Figure 1:**
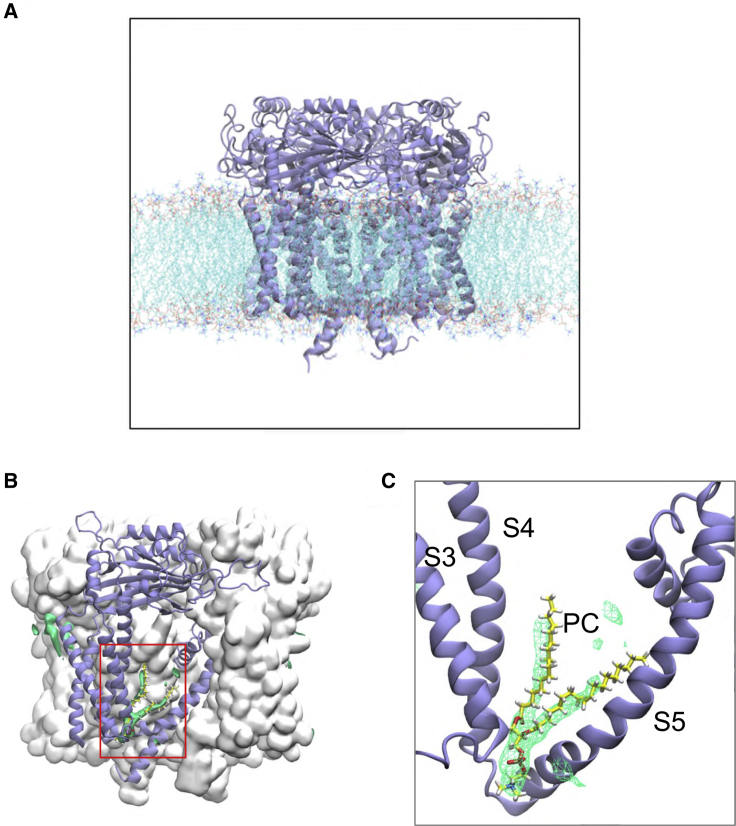
A Phospholipid Interaction Site Identified in Simulations of PC2 in a PC Bilayer (A) The PC2 channel (PDB: 5K47) embedded in a phospholipid bilayer, shown using a snapshot from an atomistic simulation of the protein (color) in a phosphatidylcholine (PC) bilayer (similar results for PDB: 5MKF and 5T4D are shown in Figure S1). The lipid tails are in cyan, phosphates in orange and red, and choline nitrogens in blue. Water molecules are omitted for clarity. (B) The PC2 protein is shown as a gray surface, viewed perpendicular to the central pore axis, with one subunit depicted as a pale purple cartoon. Green isocontour surfaces represent a high probability of occurrence of phospholipid molecules. (C) Zoomed-in view (red box in B) of the S3/S4/S5 pocket and the high phospholipid occurrence density with a PC molecule (taken from a simulation snapshot) shown within the density. See also Figure S1.

An initial exploration of possible phospholipid interaction sites on the TM domain of PC2 was made using atomistic MD simulations in which the PDB: 5K47 PC2 structure (a representative of several PC2 structures, see below) was embedded in a lipid bilayer made up of a single species of phospholipid (palmitoyl-oleyl-phosphatidylcholine [POPC]; Figure 1A). This process was repeated for all three molecular structures of wild-type PC2 (PDB: 5K47, 5MKF, 5T4D; see Figure S1) and also for a gain-of-function mutant (F604P) of PC2 (PDB: 6D1W) (Zheng et al., 2018), yielding a total of more than 2 μs of atomistic simulations of PC2 in a phosphatidyl choline (PC) bilayer (Table S1). The simulations were examined in terms of regions of high probability density of occurrence of phospholipid molecules on the protein surface. In all 12 simulations (i.e., three repeats for each of the four structures, PDB: 5K47, 5MKF, 5T4D, 6D1W), high lipid occurrence densities (Figure 1B) were observed in a pocket exposed to the intracellular leaflet of the lipid bilayer, between TM helices S3, S4, and S5 (Figure 1C), corresponding to one POPC lipid molecule bound to each subunit of the PC2 tetramer. These results are illustrated for PDB: 5K47 in Figures 1B and 1C, and similar results for PDB: 5MKF and 5T4D are shown in the Figure S1. Side chains of residues in S3, S4, and S5 create a hydrophobic pocket, within which the acyl tails of the bound lipid molecules reside. The phosphate oxygens of the bound lipid formed hydrogen bonds to the indole ring of Trp507 in S3 and to the hydroxyl group of Ser591 in the S4-S5 linker.

Thus, in a simple model lipid bilayer, we observe a phospholipid binding site on PC2 that is close to the proposed lipid and vanilloid (e.g., capsaicin) binding sites observed in cryo-EM studies of the related TRPV1 and TRPV2 channels (Gao et al., 2016). Lipid or detergent binding has been observed at a similar location in cryo-EM structures of TRPV6 (McGoldrick et al., 2018, Singh et al., 2018), TRPV5 (Hughes et al., 2018b), TRPC3 (Fan et al., 2018), TRPC4 (Vinayagam et al., 2018), TRPM4 (Autzen et al., 2018, Duan et al., 2018b), TRPML3 (Hirschi et al., 2017), and TRPM7 (Duan et al., 2018a). This site has also been suggested to be the binding site for the TRPV5 inhibitor econazole (Hughes et al., 2018a) and for PI(4,5)P_2_ (Hughes et al., 2018b). Together with our simulation results, this comparison with other TRP channels suggested that a combined experimental and computational approach to lipid interactions with PC2 was needed.

### Cryo-EM Reveals Lipid-like Density

**Figure 2:**
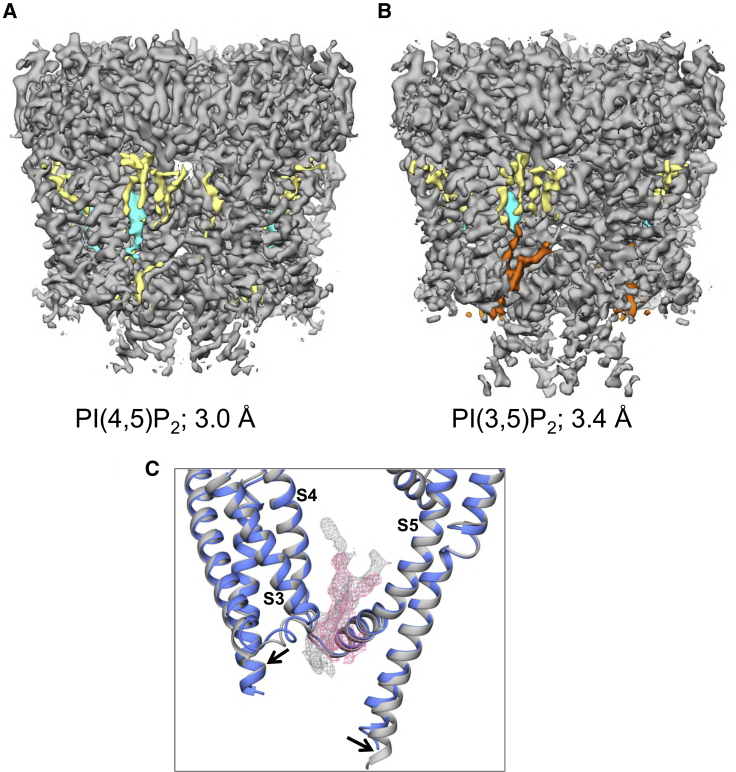
Lipid Binding Sites Suggested by cryo-EM Maps of PC2 (A and B) Lipid binding sites suggested by cryo-EM maps of PC2 obtained (A) in the presence of PI(4,5)P_2_ (3.0 Å resolution) and (B) in the presence of PI(3,5)P_2_ (3.4 Å resolution). Protein density (contoured at 3.2σ) is in gray, detergent density in yellow, lipid density in orange, and cholesterol density in cyan. (C) Expanded view around the proposed lipid binding site between S3, S4, and S5 showing density from the 3.0 Å map (pink; see Figure S9) and from the 3.4 Å map (gray). See also Figures S2, S3, and S5.

**Figure 3:**
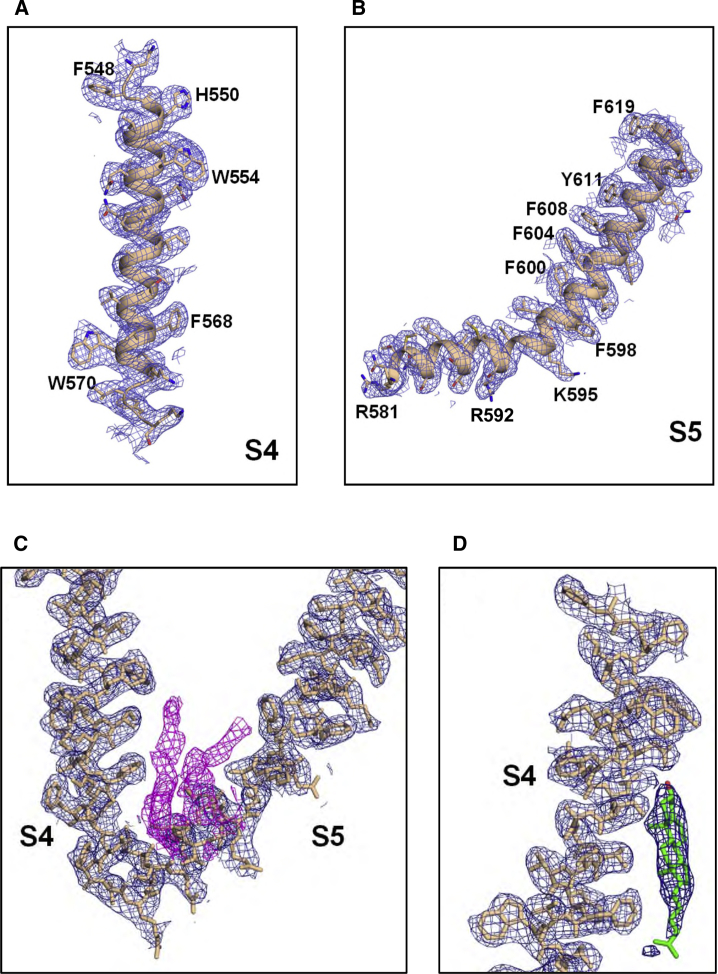
Images of the Density and Structure Relating to the Lipid Binding Sites Representative images of the density and structure relating to the lipid binding sites, from the 3.0 Å resolution cryo-EM map. (A and B) The S4 (A) and S5 (B) transmembrane helices, with aromatic and basic side chains labeled. (C) The S4 and S5 helices (yellow structure and blue density) with the density proposed to correspond to lipid (see main text for discussion) in pink. (D) The S4 helix (yellow structure and blue density) with the fitted cholesterol molecule (green structure and darker blue density; see main text and Figure 8). Density is shown filtered to 2.96 Å at 2σ. See also Figures S4 and S5.

To investigate possible binding of anionic phospholipids to PC2, we determined two cryo-EM structures of PC2: in the presence of PI(4,5)P_2_ to 3 Å resolution (Figures 2A, 3A, and 3B), and of PI(3,5)P_2_ to 3.4 Å (Figure 2B). (See Figures S2 and S3, and Table S2, for details of cryo-EM data and processing, and Figure S4 for further examples of density plus structure from the 3 Å resolution map.) We used similar conditions to our original 4.2-Å PC2 structure (PDB: 5K47), with a truncated construct (residues Pro185 to Asp723), purified in the detergent n-undecyl-β-D-maltopyranoside (UDM).

The overall conformation of the protein is the same as our original structure, i.e., a closed state at both the selectivity filter and the lower gate, although we did in this case observe a disulfide bond in the TLC1 region of the TOP domain (between residues Cys331 and Cys344; see Figure S4C). In our earlier structure (PDB: 5K47), Cys331 and Cys344 appeared to be in their reduced form with free thiol groups. In our current maps, there is clear density showing that a disulfide bond is formed between these two residues (Figure S4C), which is consistent with the 3-Å PC2 structure PDB: 5T4D (Shen et al., 2016). The two structures reported here also differ from our previous structure in that they both display a spherical density below the selectivity filter consistent with a bound ion (see Figure S4B). These densities are visible at several thresholds, and the peaks are prominent even in the un-averaged (C1) maps, which indicates that they are unlikely to be noise peaks on the symmetry axis but rather may correspond to bound cations. The identity of the bound cation is unclear as both calcium and sodium ions were present in the buffer used to prepare the EM grids. In contrast, there is only a region of weak elongated density present in the equivalent location in the 3-Å PC2 structure (EMD-8354/PDB: 5T4D).

Having observed these new features in both of the new maps, we inspected all lipid-like non-protein densities present in the TM region. In particular, we examined the region of the maps where phospholipid was found in the simulations to see whether there was any evidence for density possibly corresponding to tightly bound lipids at these sites. This revealed clear non-protein densities located between S3, S4, and S5 in both the PI(4,5)P_2 and_ PI(3,5)P_2_ maps, suggestive of lipid and/or detergent occupancy (Figures 2C, 3C, and S5). The density observed in the PI(3,5)P_2_ complex (Figure 2B) may correspond to a lipid molecule, although the presumed head group region of the density is not sufficiently well-defined to unequivocally interpret it as a PIP molecule. In the higher-resolution PI(4,5)P_2_ structure, although there is density at the same site (Figure 2A), it differs in shape and could be interpreted as two UDM detergent molecules. Although PI(3,5)P_2_ maps suggest that a lipid molecule may be bound at this site, there are few interactions between the region where the head group lies and the potential lipid. Overall, although the overall map quality is high and reveals the larger amino acid side chains (see Figures 3 and S4), for the most part the resolution in the non-protein portion is not sufficient to unequivocally assign a given lipid molecule species to this site in the final cryo-EM models, and so no lipid was placed at this site in the final PDB file. In addition to this possible lipid binding site, a region of density within the upper segment of S1–S4 helices was found in both cryo-EM maps, which exhibits clear characteristics of a UDM detergent molecule (see Figure S5A), and four other densities in the upper leaflet between S4 of one subunit and S5 and S6 of adjacent subunit were also attributed to UDM molecules. Their positions are close to the lipids/single-chain fatty acids included in the previously published structure of PC2 in amphipol (PDB: 5MKE/5MKF; Wilkes et al., 2017) (see Figure S5A). Density corresponding to a cholesterol molecule is clearly defined adjacent to S4 (Figures 2A, 2B, 3C, and S5A). This is discussed in more detail below.

In other TRP channel structures with lipid molecules built into similar locations to the potential PIP site we observed (e.g., Gao et al., 2016, Hughes et al., 2018b), the lipid head groups are coordinated not only by residues on the S3, S4, and S5 TM helices, but also by residues in the cytosolic pre-S1 or post-S6 domains. The lack of cytosolic domains in our constructs may contribute to the flexibility of the head group of the bound lipid, helping to explain why the densities observed for these structures are not well resolved in the head group region. Similarly, there is undefined density in this region in structures obtained using soy extract polar lipids (which are likely to contain some PIs) (Shen et al., 2016). We inspected the cryo-EM density map of the latter structure (https://www.emdataresource.org/EMD-8354), which was determined with PC2 in nanodiscs. In the region of interest, there is density for “well resolved” (Shen et al., 2016) lipids in the same site as in our structures, which could represent a bound phospholipid molecule.

### PC2 Interactions with PIPs

Taking together the lipid-like cryo-EM density alongside results of our initial simulations, and given the lipid interactions of other TRP channels (see above), it seemed possible that the hydrophobic pocket identified could be a binding site for anionic phospholipids. Because unambiguous experimental identification of the bound species was not possible, we returned to simulations to extend the interpretation of the experimental density.

**Figure 4:**
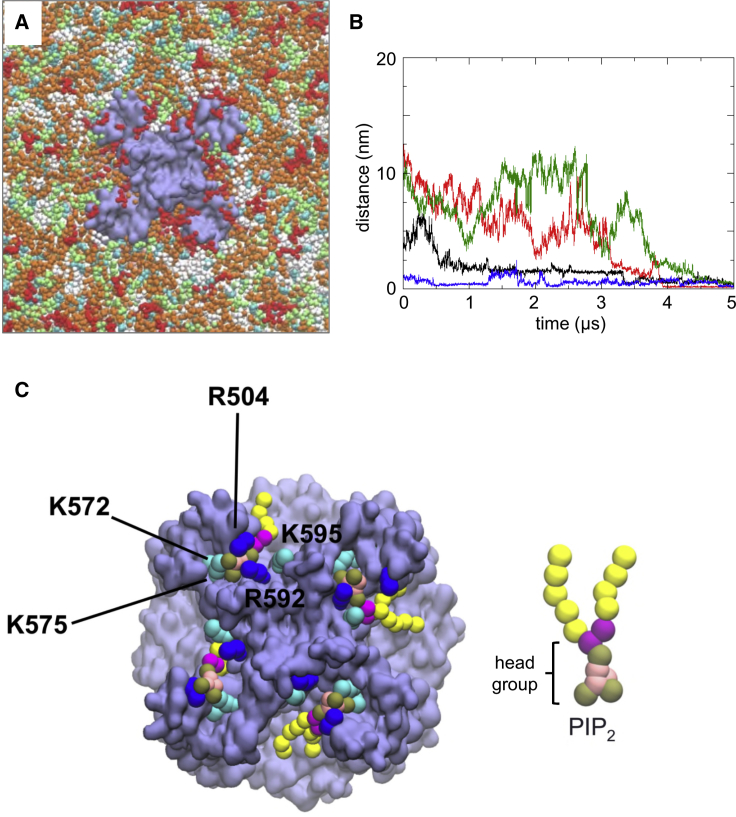
Simulations of PC2 in an *In Vivo* Mimetic Mixed Lipid Bilayer Coarse-grained (CG) simulations of PC2 in an *in vivo* mimetic mixed lipid bilayer. (A) PC2 (pale purple) in a mixed lipid bilayer, viewed from the intracellular face and showing molecules of PIP_2_ (red), cholesterol (cyan), PC (white), PE (orange), and PS (lime) in the inner leaflet of the bilayer. (B) Distance from the binding site as a function of time for four PIP_2_ molecules that bind to PC2 during a simulation in a mixed lipid bilayer. The distance is from the center of mass of the head group of each PIP_2_ molecule to the center of mass of the S505 and S591 side chains at the site to which that lipid molecule eventually binds. The four colors correspond to the four different PIP_2_ molecules that eventually bind. (C) Snapshot from the end of the simulation shown in (A) showing PIP_2_ molecules (head group phosphate particles in brown, as shown in the CG representation of PIP_2_ on the right) at four sites on the channel tetramer. Side-chain particles of basic residues at the PIP_2_ binding site are shown in blue (arginine) and cyan (lysine). See also Figures S6 and S7.

We used coarse-grained (CG) simulations of PC2 in a lipid bilayer containing multiple lipid species (Ingolfsson et al., 2014, Koldsø et al., 2014) to explore the possible specificity of the phospholipid binding site. We embedded PC2 in an *in vivo* mimetic bilayer (Figure 4A), which contained the anionic lipids phosphatidylserine (PS) and PIP_2_ in the inner leaflet, a glycolipid (GM3) in the outer leaflet, and cholesterol (CHOL) in both leaflets of the bilayer. The lipid composition of the *in vivo* mimetic bilayer membrane provided an approximation to the major lipid species likely to be present within a mammalian cell membrane (Koldsø et al., 2014, Sampaio et al., 2011). Thus, the outer (i.e., extracellular or organelle luminal) leaflet contained PC:PE:SM:GM3:CHOL = 40:10:15:10:25; and the inner (i.e., intracellular) leaflet contained PC:PE:PS:PIP_2_:CHOL = 10:40:15:10:25. This provides an overall PIP_2_ concentration of 5%, which is within the physiological range for a mammalian cell membrane (Sampaio et al., 2011, van Meer et al., 2008).

Three independent simulations (each of 5 μs duration) of a single PC2 channel (structure PDB: 5K47) inserted in an *in vivo* mimetic lipid bilayer with different random distributions of lipid molecules were performed. Simulations were also performed for the constitutively active F604P PC2 mutant (PDB: 6D1W; see Table S1). In simulations, PIP_2_ molecules diffused in the bilayer on a timescale of microseconds (Figures 4B and S6; Movie S1) resulting in random encounters with the channel molecule followed by binding to the previously identified sites on PC2, as demonstrated by tracking the distance versus time of the PIP_2_ molecules from their eventual binding sites (Figure 4B). From a final snapshot of one such simulation (Figure 4C), it can be seen that a PIP_2_ molecule has bound to each of the four sites on PC2. The head groups of the PIP_2_ molecules each interact with up to five basic residues in the S3/S4/S5 region: Arg504, Lys572, Lys575, Arg592, and Lys595. These residues are highly conserved in PC2 from different species (Figure S7), and this interaction is persistent in both wild-type and mutant PC2. Given the presence of other negatively charged lipid species (PS) in the bilayer and of positively charged patches on the protein surface, the observation that PIP_2_ molecules were able to bind to the intracellular binding site suggests that this site may be specific for PIP_2_ and related lipids.

Movie S1 Coarse Grained Simulation of the Interaction of PIP2 Molecules (Yellow/Bronze/Pink) with a PC2 channel (Pale Purple) Viewed from the Cytoplasmic Side of the Membrane. Amino Acid Sidechains at the Binding Sites are in Blue and Cyan

### Free Energy Landscapes for PC2/Phospholipid Interactions

**Figure 5:**
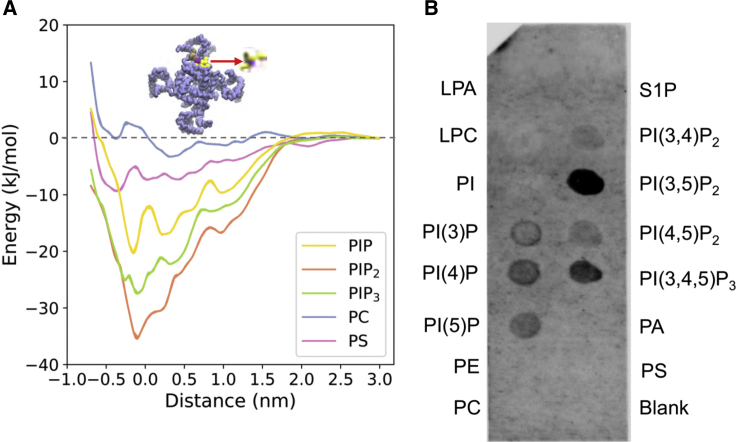
Free Energy Landscape for Lipid Interactions Free energy landscape for lipid interactions at the binding site on PC2. (A) Potentials of mean force of the interactions of PIP_2_ (orange), PIP_3_ (green), PI(4)P (yellow), PS (purple), and PC (blue) with the lipid binding site on PC2. The inset shows a schematic representation of the reaction coordinate corresponding to the distance between centers of mass of the head group of a lipid molecule and of the two serines (S505 and S591) within the lipid binding sites. (B) PIP strip data for interactions of different phospholipid species with PC2. Note the null results for the anionic PA, PS, and S1P lipids, and for the zwitterionic PE and PC controls. See also Figure S8.

To explore the possible selectivity of the PIP_2_/intracellular site interaction in more detail, we calculated potentials of mean force (PMFs) based on CG simulations. A PMF provides a one-dimensional free energy landscape for lipid/PC2 interactions, and allows us to explore the specificity of this interaction (Figure 5A). This approach has been used to explore interactions of anionic lipids (e.g., cardiolipin and PIP_2_) with a number of transporters (e.g., ANT1[Hedger et al., 2016]), ion channels (e.g., Kir channels [Domański et al., 2017]), and receptors (e.g., class A GPCRs [Song et al., 2019, Yen et al., 2018]).

To estimate and compare the free energy landscapes for phosphatidylinositol monophosphate (PIP), PIP_2_, phosphatidylinositol trisphosphate (PIP_3_), PC, and for PS, each interacting with PC2 at the intracellular site defined by S3/S4/S5, CG models of a PC2 channel molecule embedded in a PC bilayer with a PIP, PIP_2_, PIP_3_, or PS molecule inserted into each of the four binding sites were used. For computational efficiency, the PC2 structure was truncated, removing the TOP domain (Ser244–Leu462). The initial configurations were lipid bound states. One of the bound lipid molecules was pulled away from the binding site to assess the free energy of interaction. A one-dimensional reaction coordinate was defined as the distance, in the plane of the bilayer, between the centers of mass of the lipid head group and two serines (Ser505 and Ser591) within the lipid binding site of PC2. The free energy profile (or PMF) for PIP_2_ shows a clear minimum close to the initial position of the PIP_2_ molecule in the binding site, with a well depth of −37 kJ/mol (Figure 5A). This is comparable with the well depth for PIP_2_ binding to the Kir2.2 channel (Domański et al., 2017). Comparison of PMF profiles as a function of window simulation duration suggested that convergence had been achieved by 1.5 μs per window (see Figure S8) and that errors on the well depths of the PMFs are of the order of ±3 kJ/mol (see Figure S8C). PIP and PIP_3_ exhibited weaker binding to PC2 with free energies of –20 and –28 kJ/mol, respectively. To confirm this, we performed CG free energy perturbation simulations (Corey et al., 2019), which suggested *ΔΔG*s of +21 and +4 kJ/mol for the conversion of bound PIP_2_ to PIP and PIP_3_ respectively, which is consistent with the pattern of selectivity derived from the PMFs (from which the corresponding *ΔΔG*s are +17 and +9 kJ/mol). We note that, due to the limitation of the CG model, we were unable to distinguish between PIP(4,5)P_2_ and PI(3,5)P_2_ interactions by calculation of CG PMFs. However, the simple anionic lipid PS showed significantly weaker binding to PC2 with a well depth of approximately –9 kJ/mol, indicating a clear selectivity of the binding site for PIP species over PS. PC shows even weaker interactions, as would be anticipated given the PMF is evaluated for a single lipid molecule in an environment of a PC bilayer. The two minima in the PMF for the PC are likely to reflect annular shells of relatively immobilized lipid molecules around the PC2 channel, as have been seen for a number of channels and other membrane proteins in simulations (Goose and Sansom, 2013, Niemela et al., 2010).

### Testing the Predicted Lipid Specificity

The predicted binding of PIP_2_ to PC2 was tested biochemically using PIP strips (Shirey et al., 2017), nitrocellulose membranes with lipids including phosphoinositides and other phospholipids spotted onto their surface. Protein binding to relevant lipids can be detected with antibodies to the tagged protein. This method has been used, for example, to confirm binding of PIP_2_ to the GluA1 ionotropic glutamate receptor (Seebohm et al., 2014). The results from this assay (Figure 5B; N = 5 biological repeats; see Figure S9) suggest that truncated PC2 in UDM micelles can bind a range of PIPs, including PIP_2_ species. However, variation between the biological repeats (see Figure S9) means we were not able to determine whether, e.g., PIP_2_ or PIP_3_, binds more tightly. In contrast, PC2 does not exhibit interactions with simple anionic (PS, PA, and SP1) or zwitterionic (PE and PC) lipids.

### A PIP_2_ Binding Site

Having established that PC2 binds PIP_2_ selectively, we examined the interactions of the protein with the lipid in more detail, based on a CG simulation snapshot structure of the PC2-PIP_2_ complex corresponding to the energy minimum in the PMF. As noted above, the head group of PIP_2_ interacts with five basic residues: Arg504, Lys572, Lys575, Arg592, and Lys595. In particular, the 1′-phosphate interacts closely with Arg592, and the 4′- and 5′-phosphates interact with Lys572 and Lys575, respectively. Comparable binding sites for PIP_2_ were formed by clusters of basic residues as seen in other ion channels, e.g., Kir channels (Hansen et al., 2011, Lacin et al., 2017), and GPCRs (Song et al., 2019, Yen et al., 2018).

**Figure 6:**
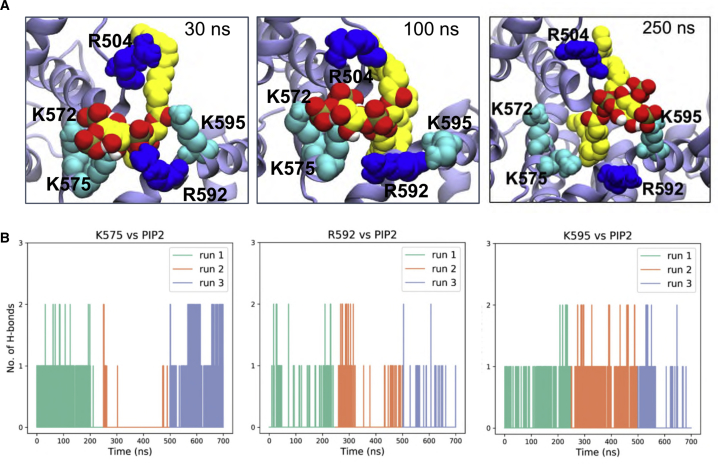
Protein-Lipid Interactions of the Head Group of PIP_2_ Protein-lipid interactions showing the head group of PIP_2_ coordinated by five basic residues (R504, K572, K575, R592, and K595) at the binding site. (A) Snapshot structure corresponding to the CG energy minimum was converted into an atomistic representation and used as the basis of 30 ns distance restrained simulations (see text for details) followed by 250 ns of unrestrained simulations (with snapshots at 100 and 250 ns). (B) H-bonding interactions between key basic side chains and the PIP_2_ head group during three replicates (run 1, 0–250 ns; run 2, 250–500 ns; run 3, 500–700 ns) of the unrestrained atomistic simulations.

To evaluate the proposed PIP_2_ binding site in more detail, a CG structure corresponding to the free energy minimum was converted into an atomistic representation. A two-stage atomistic simulation was then performed. Firstly, a short (30 ns) simulation was performed in which harmonic restraints were applied to the distances between the 1′-, 4′-, and 5′-phosphates of PIP_2_ and the side chains of Arg592, Lys575, and Lys572, respectively, in order to relax the atomistic model while maintaining the interactions seen in the CG PMF calculations. The distance restraints were then removed and three replicates of an unrestrained simulation (durations 200–250 ns) were run to allow the PIP_2_ to explore more fully the binding site on PC2. Final snapshots from the restrained and unrestrained simulations are shown (Figure 6A). The interactions between the lipid head group and the interacting residues are seen to be dynamic and to vary stochastically between replicate simulations. Thus, for residues Arg504, Lys575, Arg592, and Lys595, fluctuating numbers of hydrogen bonds were formed with PIP_2_ throughout the simulations (Figure 6B; Movie S2). Such dynamic fluctuations in the interactions of bound PIP_2_ are not unique to PC2; for example, they have also been reported for PIP_2_ molecules bound to Kir channels (e.g., Lacin et al., 2017).

Movie S2 Simulation Showing the Flexible Nature of the Interactions of a PIP2 Molecule with its Binding Site on PC2. Colours as for Movie S1.

### PIP Interactions in TRP Channels

**Figure 7:**
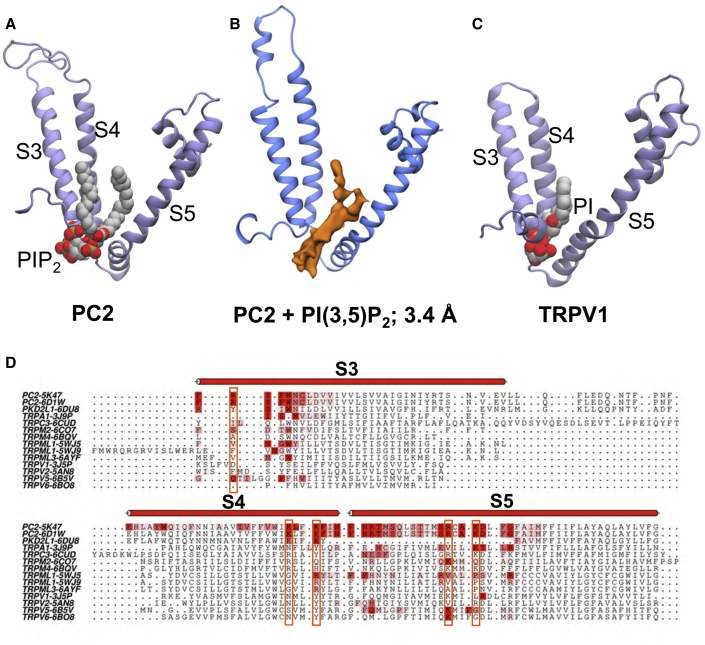
Comparison of PI Lipids Bound to PC2 and to TRPV1 Comparison of PI lipids bound to PC2 and to TRPV1 with cryo-EM density. (A–C) (A) PIP_2_ bound to PC2 (as revealed by the current simulation study); (B) lipid-like density in the cryo-EM maps of PC2 obtained in the presence of PI(3,5)P_2_ (3.4 Å resolution; see Figure 5); (C) PI bound to TRPV1 (as revealed by cryoelectron microscopy, PDB ID 5IRZ). In each case the lipid molecule or density is located between the S3, S4, and S5 helices of the VSLD. (D) A sequence alignment colored on contacts with PIP_2_ in the mixed lipid simulations. Residues of the region around the binding pocket between the S3, S4, and S5 helices are colored (on a white to red scale) based on the mean duration of the interactions of PIP_2_ head groups with each residue. The five basic residues of PC2 which form interactions with the head group of PIP_2_ at the binding site (i.e., R504, K572, K575, R592, and K595) are boxed. See also Figure S5.

The proposed PIP interaction site on the TM domain of PC2 may be compared with a lipid binding site, close to the vanilloid ligand-binding site, which has been seen in the structure of TRPV1 and has been interpreted as corresponding to a bound phosphatidylinositol molecule (Gao et al., 2016). Comparison of the two sites (Figures 7A and 7C) reveals strong similarities, especially the location of the anionic phosphate-containing lipid head group at the N-terminal region of the S3 helix dipole (Hol et al., 1978). Furthermore, the PIP_2_ interaction site on PC2 and the PI site on TRPV1 both agree well with the lipid-like density in our 3.4-Å cryo-EM map (Figure 7B). To explore this possible common binding site further we extended our CG simulations in a mixed lipid *in vivo* mimetic to 12 different TRP channel structures (see Table S1). For each of the TRP channel structures we then analyzed the mean contact duration of each residue with the head group of a PIP_2_ molecule over the course of the simulation. These contact durations ranged up to >1 μs (e.g., Figures 4B and S6). The results (Figure 7D) revealed a degree of conservation of the proposed PIP channel interaction between different families of TRP channels. In particular, an aromatic contact in S3 and basic contacts in S4 and S5 are also seen for TRPML2, TRPML1, TRPML3, and, to a lesser extent, in TRPV5 and TRPV6.

Returning to the cryo-EM data, we noted that with the more soluble version of PIP_2_ (i.e., PI(3,5)P_2_), we observed in the map a small outward movement (∼2 Å; see Figure S5A) of the VSLD at the S2–S3 linker region and an inward movement (∼2 Å) toward the central axis of the cytosolic extension of S6 helix in the PI(3,5)P_2_ structure compared with the PI(4,5)P_2_ structure (which may correlate with the apparently stronger binding of PI(3,5)P_2_ seen in the PIP strip assay). This is suggestive of an interplay between occupancy of the PIP_2_ site and the interactions between the VSLD and pore domains. However, we note that our structures are of a truncated construct of PC2, and it is possible that the missing intracellular domain(s) may be needed to observe a more extensive change in conformation.

### Cholesterol Interactions

**Figure 8:**
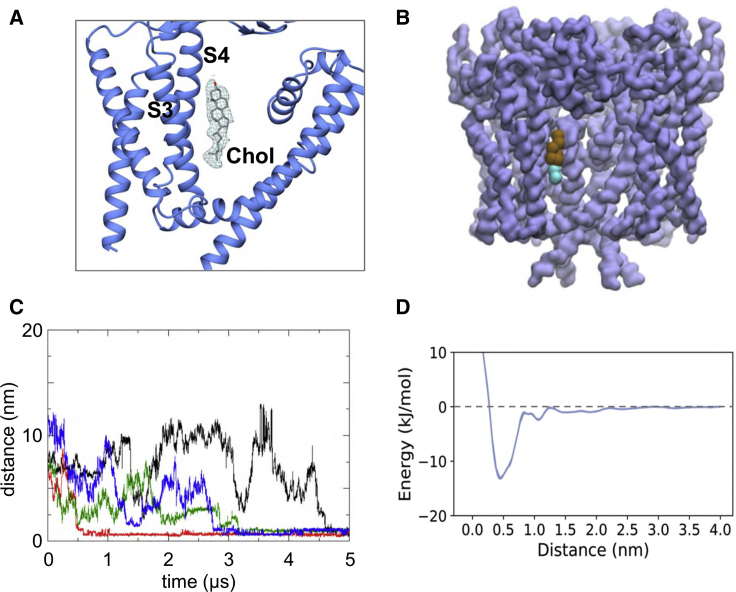
Cholesterol Interactions with PC2 (A) Cryo-EM density (from the 3.0 Å map, contoured at 2.2σ; see Figure 2A) corresponding to a binding site for cholesterol located between the S3 and S4 helices and helix S6 of the adjacent subunit. (B) Cholesterol observed to bind to the same site in CG simulations of PC2 in an *in vivo* mimetic mixed lipid bilayer (see Figure 3). (C) Distance from the binding site as a function of time for four cholesterol molecules which bind to PC2 during a simulation in a mixed lipid bilayer. (D) Potential of mean force for the interaction of cholesterol with the binding site on PC2. See also Figures S5 and S10.

Further examination of the cryo-EM density also revealed a possible site for (co-purified) cholesterol (Figure 8A; see also Figures 3C and S5A) located between the S3 and S4 helices of the VSLD and pore domain S6 helix of the adjacent subunit. The cholesterol must have remained bound to PC2 throughout the extraction and purification process, since neither cholesterol, nor the cholesterol mimetic, cholesteryl hemi-succinate (CHS), was used in the PC2 purifications. Interestingly, comparable density is also visible in the cryo-EM maps for the PDB: 5T4D structure, consistent with the presence of cholesterol in this structure (Shen et al., 2016).

Given the importance of cholesterol in ciliary membranes (Garcia et al., 2018), we analyzed our CG-MD simulations of PC2 in an *in vivo* mimetic (i.e., mixed lipid, see above and Table S1) bilayer to identify possible interactions of cholesterol with the channel (Figures 8B, 8C, and S10A). These simulations indicated a cholesterol interaction site corresponding to that revealed by the cryo-EM density. This indicates that this site is likely to interact with cholesterol present in the bilayer. Having identified a cholesterol binding site, we performed CG PMF calculations to estimate the free energy landscape for the PC2-cholesterol interaction (Figure 8D). These calculations showed a weaker interaction than for PIPs (above), with a free energy well depth comparable with that for binding of cholesterol to other membrane proteins (e.g., Hedger et al., 2019).

**Figure 9:**
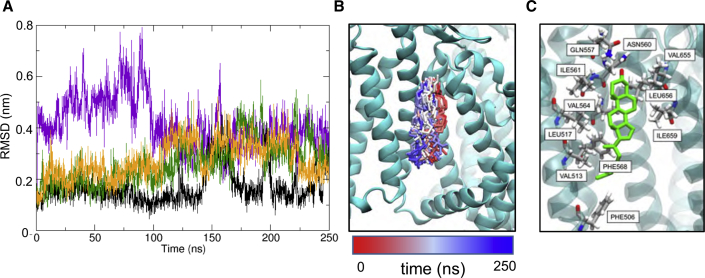
Simulations of Cholesterol-Bound PC2 Atomistic simulations of cholesterol-bound PC2, with the cholesterol molecule initially built into the cryo-EM density. (A) Root-mean-square deviation (RMSD) versus time for the four cholesterol molecules bound to PC2 during a 250-ns atomistic MD simulation. The large fluctuations in RMSD for two molecules (purple and green curves) demonstrate the relatively loose binding of cholesterol at this site. (B) Snapshot structures of cholesterol (stick representation; taken every 1 ns) at the binding site on PC2. Each of these structures is colored according to the corresponding simulation time on the red, white, and blue color scale shown; thus structures at the start of the simulation are colored red, and those at the end in blue. (C) Final simulation snapshot (t = 250 ns) showing the arrangement of key binding site residues. See also Figure S10.

To further evaluate the cholesterol binding site, we performed atomistic simulations (3 × 250 ns) starting from coordinates for cholesterol built into the cryo-EM density. From the simulations it is evident that cholesterol interacts dynamically at this site, which is located at the interface between the VSLD and S6, with the long axis of cholesterol running approximately parallel to helices S3 and S4. Thus, for the four symmetry-related binding sites on the PC2 channel, one site exhibits stably bound cholesterol, one site has a cholesterol that transiently dissociates then rebinds, and the two other sites show intermediate behavior (noting that these differences are simply the stochastic dynamics of a single molecule simulation; Figures 9A and S10B). This is consistent with, e.g., simulation studies of cholesterol on GPCRs (see Hedger et al., 2019 for a detailed discussion), which indicate relatively dynamic, loose binding as is also seen for PC2 (Figure 9B). The hydroxyl group of cholesterol mainly hydrogen bonded to Gln557 and Asn560. The steroid nucleus of cholesterol sits within a shallow hydrophobic pocket formed by a group of isoleucine (Ile561 and Ile659), leucine (Leu517 and Leu656), and valine (Val564 and Val655) residues (Figure 9C). The hydrocarbon chain of cholesterol showed considerable mobility throughout the simulation, which may explain the lack of density around this region in our lower resolution cryo-EM map.

### Conclusions

Using an approach combining multiscale MD simulations and cryo-EM, we identified a phospholipid binding pocket in the human TRP channel PC2 between the S3 and S4 TM helices and the S4-S5 linker. Free energy calculations suggested that this binding site is selective for PIP_2_ and related PIPs, and this is supported by biochemical data. The location of this site is analogous to the shared binding site for vanilloid ligands and phosphatidylinositol lipids in the canonical TRPV1 channel (Figures 7 and S5B). This region is distinct from the location between the VSLD and the core pore-forming domain of the PDB: 5MKE structure of PC2 (Wilkes et al., 2017) where lipid-like density is modeled as phosphatidic acid and as palmitic acids binding within the extracellular leaflet region of the channel protein (see Figure S5A).

We should consider possible limitations of this study. The resolution currently achievable in cryo-EM studies of membrane proteins means that it is sometimes difficult to unambiguously identify bound lipids (however, see Laverty et al., 2019, for a counterexample where lipid identification proved possible). In particular, certain regions of cryo-EM have lower resolution than other regions of the maps. The regions containing the lipids, usually on the outer surface of the protein, are often of lower resolution, making it more challenging to determine the nature of the bound lipids. Molecular simulations thus provide a valuable tool in assessing possible identities of bound lipids. In our studies, MD simulations are strongly suggestive of PIP_2_ and cholesterol binding at the two sites identified on PC2. However, the cryo-EM data only allowed unambiguous confirmation of these predictions in the case of cholesterol, not PIP_2_. PIP strip assays were used to identify possible phospholipid species interacting with PC2, but quantification of relative binding affinities for different lipids could not be established. In our PMF calculations, coarse graining of the lipid models does not allow distinction between different PIP species with the same charge. In addition, the PC2 construct used for PIP strips is truncated (as is the construct in the structural studies and the simulations). Therefore, it is uncertain whether full-length PC2 protein would exhibit exactly the same lipid binding behavior, especially for the PIP binding site which is close to the termini of the truncation.

**Figure 10:**
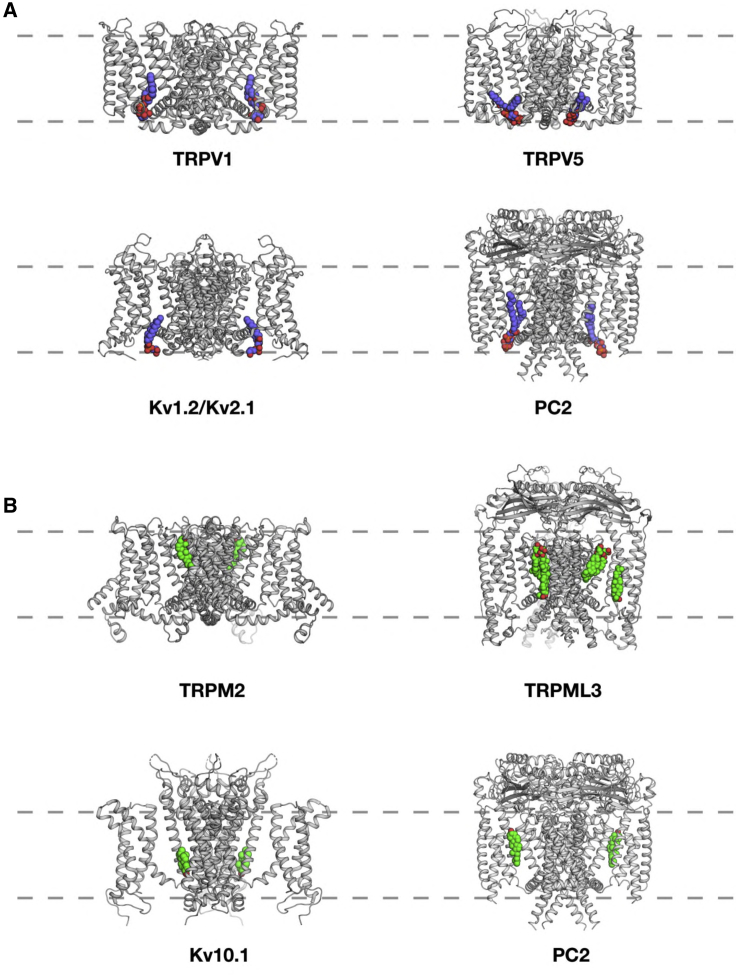
Comparison of Lipid Binding Sites of TRP and Kv Channels (A) Anionic lipid (blue acyl tails and red phosphate oxygens) binding sites close to the S4 linker: TRPV1 and PI (PDB: 5IRZ), TRPV5 and PIP_2_ (PDB: 6DMU), Kv1.2/Kv2.1 chimera and PG (PDB: 2R9R), and PC2 and PIP_2_ (this study). (B) Cholesterol (green carbons and red oxygens) binding sites between the VSD/VSLD and the central pore domains of: TRPM2 (PDB: 6CO7), TRPML3 (PDB: 5W3S), Kv10.1 (PDB: 5K7L), and PC2 (this study). In each case the transmembrane region of the channel structure is shown, with the location of the lipid bilayer shown via the broken gray lines.

Comparison with other ion channels and their lipid interactions suggest that the PIP_2_-binding site on PC2 may be of functional importance. A number of TRP and related channels show anionic lipid interactions in this region, close to the S4-S5 helix which links the VLSD/VSD to the pore domain. For example, TRPV1-PI, TPRV5-PIP_2_, and Kv1.2/2.1-anionic lipid interactions all occur at similar sites to the PC2-PIP_2_ interaction (Figure 10A). PIP_2_ regulates a number of TRP channels (as discussed above) and also a number of Kv channels (Kruse et al., 2012) via interactions with the S4-S5 linker. This suggests that the PC2 PIP_2_-binding site near the S4-S5 linker may be a regulatory/allosteric site. Interestingly, some pathogenic missense variants (see http://pkdb.mayo.edu/) in PC2 occur in the vicinity of the PIP_2_ site, e.g., L517R, D511V, and N580K (the latter located at the start of the S4-S5 linker), and might be expected to perturb interactions with PIP_2_.

Structural and simulation studies have shown that the movement of the S4-S5 linker is important for gating in voltage-gated cation channels. For example, in the voltage-gated potassium channel Kv1.2, the constriction or dilation of the pore domain is controlled by the voltage sensor domain through the S4-S5 linker (Long et al., 2005). The TM helices of TRP channels and voltage-gated cation channels share high structural similarity, indicating a possible common functional role of the S4-S5 linker. Indeed, it has been shown that TRPV1 (Gao et al., 2016, Yang et al., 2015), TRPV2 (Yang et al., 2016), and TRPV4 (Teng et al., 2015) use the same mechanism to gate the channels, and disruption of the interaction configurations at the linker region by ligands/lipids could change the gating probability.

It has been suggested that the basal regions of primary cilia membranes contain PIP_2_ while the upper regions of the ciliary membrane have an elevated level of PI(4)P (Garcia et al., 2018, Nakatsu, 2015). Thus, it is reasonable to suppose that PC2 binds to PIP_2_ *in vivo*. In this context it is of interest that OCLR1, a lipid phosphatase that converts PI(4,5)P_2_ to PI(4)P, modulates the length of cilia in renal epithelial cells, and that loss of its function in Lowe syndrome is associated with progressive renal malfunction (Rbaibi et al., 2012).

Our structural and simulation data also reveal a cholesterol binding site on the outer-leaflet-facing surface of the PC2 molecule. This seems to be close to the site suggested to interact with a phospholipid (modeled as phosphatidic acid; see PDB: 2MKF and 2MKE) in the study of Wilkes et al. (2017). It is distinct from but adjacent to the sites interpreted as corresponding to CHS in the latter study. Examination of structures (Figure 10B) of a number of other TRP channels (e.g., TRPM2 and TRPML3) and more distantly related channels (e.g., Kv10.1) reveals multiple cholesterol interaction sites between the VSLD/VSD and the pore domain. Although interactions of TRP channels with cholesterol have not been studied in detail, some physiological data are available (Morales-Lazaro and Rosenbaum, 2017). For PC2, mutations of two of the residues forming the cholesterol binding site (Leu517Arg and Leu656Trp) are classified as “likely pathogenic” and “likely hypomorphic,” respectively (see http://pkdb.mayo.edu). It is tempting to suggest the likely biological importance of cholesterol interactions with PC2. For example, cholesterol plays a key role in signaling via the ciliary GPCR Smoothened, and it is possible that different regions of cilia differ in the cholesterol content of their membranes (Luchetti et al., 2016). However, further biochemical studies are needed to establish a functional role of cholesterol in regulation of PC2 in ciliary membranes. It is also interesting to note that pregnenolone sulfate has been identified as an activator of PC2, in the presence of other TRP channels (Kleene et al., 2019). Given the similarity between the structures of cholesterol and pregnenolone, we speculate that the two molecules could occupy the same binding site, placing the sulfate on the extracellular surface, near the pore, thus activating the channel. Given the emerging importance of cholesterol interactions with other ion channels and with receptors (Bukiya and Rosenhouse-Dantsker, 2017, De Jesus-Perez et al., 2018, Duncan et al., 2019, Lee, 2018) it would not be surprising if cholesterol interactions with TRP and related channels were of functional importance.

Overall, the structural and simulation results presented here suggest that further studies of possible functional effects of PIPs and/or of cholesterol on PC2 channels are merited. From a broader perspective, this study demonstrates that cryo-EM and MD together provide a powerful combination for revealing lipid interactions of ion channels, enabling identification of the molecular identity and interactions of lipid-like densities observed in structures. This is of particular importance when the resolution of cryo-EM maps is such that possible lipid molecules are difficult to identify unambiguously. Our results help to establish that the interactions of TRP channels with lipids will enable definition of novel druggable sites on this physiologically important class of channel molecules.

## STAR★Methods

### Key Resources Table

**Table:** 

REAGENT or RESOURCE	SOURCE	IDENTIFIER
**Chemicals, Peptides and Recombinant Proteins**		

n-Dodecyl-β-D-Maltopyranoside (DDM)	Anatrace	Cat# D310S
n-Undecyl-β-D-Maltopyranoside (UDM)	Anatrace	Cat# U300LA
Sf-900™ II SFM	Thermo Fisher Scientific	Cat# 10902088
Insect-XPRESSTM Protein-free Insect Cell Medium	Lonza	Cat# BE12-730Q
α-L-Fucosidase from bovine kidney	Sigma-Aldrich	Cat# F5884
Bovine Serum Albumin	Sigma-Aldrich	Cat# A7030
DYKDDDDK Tag Monoclonal Antibody (FG4R), HRP	Thermo Fisher Scientific	Cat# MA1-91878-HRP; RRID: AB_2537626
18:0-20:4 PI(4,5)P_2_	Avanti Polar Lipids, Inc.	Cat# 850165
18:1 PI(3,5)P_2_	Avanti Polar Lipids, Inc.	Cat# 850154

**Critical Commercial Assays**		

Superose 6 Increase 10/300 GL	GE Healthcare	Cat# 29-0915-96
PIP Strips™ Membranes	Thermo Fisher Scientific	Cat# P23751
TALON® Metal Affinity Resin	Clontech Laboratories, Inc.	Cat# 635504

**Bacterial and Virus Strains**		

MAX Efficiency DH10Bac™	Thermo Fisher Scientific	Cat# 10361012

**Deposited Data**		

TRPV1 structure	(Gao et al., 2016)	PDB: 5IRZ
TRPV5 structure	(Hughes et al., 2018b)	PDB: 6DMU
Kv1.2/Kv2.1 structure	(Long et al., 2007)	PDB: 2R9R
TRPM2 structure	(Zhang et al., 2018)	PDB: 6CO7
TRPML3 structure	(Hirschi et al., 2017)	PDB: 5W3S
Kv10.1 structure	(Whicher and MacKinnon, 2016)	PDB: 5K7L
Coordinates of human PC2 with 18:0-20:4 PI(4,5)P_2_	This study	PDB: 6T9N
Coordinates of human PC2 with 18:1 PI(3,5)P_2_	This study	PDB:6T9O
Cryo-EM map of human PC2 with 18:0-20:4 PI(4,5)P_2_	This study	EMDB: 10418
Cryo-EM map of human PC2 with 18:1 PI(3,5)P_2_	This study	EMDB: 10419

**Experimental Models: Cell Lines**		

*Spodoptera frugiperda* (Sf9) insect cells	Thermo Fisher Scientific	Cat# 11496015

**Recombinant DNA**		

Human PC2: 185-723 cloned into the expression vector pFBCT10HF-LIC	Addgene	Plasmid #98226

**Chemicals, Peptides, and Recombinant Proteins**		

Primer:hPC2-185 Forward: TTAAGAAGGAGATATACTATGCCCCGAGTG GCCTGGGCG	Eurofin Genomics	N/A
Primer:hPC2-723 Reverse: GATTGGAAGTAGAGGTTCTCTGCATCCACG GTATTTTTTT TCAGT	Eurofin Genomics	N/A

**Software and Algorithms**		

GROMACS	Abraham et al., 2015	www.gromacs.org
VMD	Humphrey et al., 1996	https://www.ks.uiuc.edu/Research/vmd/
PyMOL	DeLano, 2002	https://pymol.org/2/
MotionCor2	Zheng et al., 2017	https://emcore.ucsf.edu/cryo-EM-software
RELION 3	Kimanius et al., 2016	https://www3.mrc-lmb.cam.ac.uk/relion/index.php?title=Main_Page
CTFFIND-4.1	Rohou and Grigorieff, 2015	http://grigoriefflab.janelia.org/ctffind4
Gautomatch	http://www.mrc-lmb.cam.ac.uk/kzhang/	http://www.mrc-lmb.cam.ac.uk/kzhang/
COOT	Emsley and Cowtan, 2004	https://www2.mrc-lmb.cam.ac.uk/personal/pemsley/coot/
UCSF Chimera		https://www.cgl.ucsf.edu/chimera/
PHENIX	Adams et al., 2010	https://www.phenix-online.org/
RESMAP	Kucukelbir et al., 2014	http://resmap.sourceforge.net/
Molprobity		http://molprobity.biochem.duke.edu/

### Lead Contact and Materials Availability

Further information and requests for resources and reagents should be directed to and will be fulfilled by the Lead Contacts, Mark Sansom (mark.sansom@bioch.ox.ac.uk). All unique/stable reagents generated in this study are available from the Lead Contact with a completed Materials Transfer Agreement.

### Experimental Model and Subject Details

#### Cell Culture for Protein Expression

PC2 proteins (a truncated stable construct, hPC2: Pro185 - Asp723) was expressed and purified from baculovirus infected *Spodoptera frugiperda* (Sf9) insect cells, grown in in Sf900II serum free, protein-free insect cell medium with L-glutamine (Thermo Fisher Scientific).

### Method Details

#### Simulation Model Preparation

N-acetyl-D-glucosamines were removed from all protein structures, and missing side chains and loops were modelled using MODELLER version 9v10 (Fiser and Sali, 2003). Models were visually inspected. Structurally realistic models with the lowest value of the MODELLER objective function were chosen for subsequent simulations.

#### CG Simulations

CGMD simulations were performed using GROMACS version 4.6 (www.gromacs.org) (Pronk et al., 2013) with the MARTINI version 2.1 force field (Monticelli et al., 2008). CG simulations within PMF calculations were done with GROMACS version 5.1 (www.gromacs.org) (Abraham et al., 2015) with the MARTINI version 2.2 force field (de Jong et al., 2013). An elastic network (Periole et al., 2009) was applied with elastic bond force constant of 500 kJ/mol/nm^2^ and an upper elastic bond cut-off of 0.9 nm. The standard MARTINI water beads with van der Waals radii of 0.21 nm were used to solvate all systems, which were then neutralised with NaCl at a concentration of 0.15 M. CG lipids and ions were described by MARTINI version 2.0 lipids and ions respectively.

Each simulation system contains only one copy of a protein. Initially, PC lipids were randomly placed around the proteins. Correct positioning of protein in a lipid bilayer was achieved by a 100 ns self-assembly simulation (Scott et al., 2008). The CG system for PDB ID: 5K47 was equilibrated for 400 ns after the initial self-assembly. Simulation systems for the other PC2 structures (i.e. PDB ID: 5MKF and 5T4D) were set up by aligning and replacing the protein in the PDB 5K47 POPC-only system, and then equilibrating for 1 μs. Systems used for mixed-lipid simulations were set up by exchanging PC molecules for other lipids using a locally developed script.

All CGMD simulations were performed at a temperature of 310 K and a pressure of 1 bar. V-rescale thermostat (Bussi et al., 2007) was used to maintain the temperature using a coupling time constant of τ_t_ = 1 ps. Protein, lipids, and solvent (water + ions) were coupled separately to the temperature bath. For self-assembly and equilibration simulations, pressure was controlled by semi-isotropic pressure coupling with the Berendsen barostat (Berendsen et al., 1984) with a compressibility of 5 × 10^−6^/bar and a time constant of τ_p_ = 4 ps. Production runs used a Parrinello-Rahman barostat with coupling constant of 12 ps and compressibility of 3 × 10^−4^/bar for pressure control.

#### Atomistic Simulations

These were setup and run using a two-step multiscale procedure (Stansfeld et al., 2015, Stansfeld and Sansom, 2011), starting with a CG simulation to stably insert the protein in a lipid bilayer (see above), followed by conversion to an atomistic representation and subsequent atomistic simulations. A fragment based protocol (Stansfeld and Sansom, 2011) was used for the CG to atomistic conversion. For the PDB 5K47 system in a PC-only bilayer, the final frame of the 400 ns equilibration was converted to atomistic representation. Three repeats of 200 ns atomistic simulations with different initial random seeds were performed after a 6 ns unrestrained equilibration. Simulations were carried out using GROMACS version 4.6 with the CHARMM36 force field (Best et al., 2012). The water model used was TIP3P (Jorgensen et al., 1983). Atomistic systems for PDBs 5MKF and 5T4D were converted from the final frame of the 1 μs CG equilibration runs. Three repeats of 250 ns atomistic simulations were done for both structures after 1ns fully restrained equilibration.

Simulation systems of PC2 structures with bound cholesterol were setup using the CHARMM-GUI (www.charmm-gui.org). The protein with bound cholesterols were embedded in POPC-only bilayer. Simulations were performed using GROMACS version 5.1 with the CHARMM36m (Huang et al., 2017) force field and TIP3P water model (Jorgensen et al., 1983). Equilibration of the system was done in six steps with gradually decreasing restraint force constants on the protein and cholesterol molecules. Three repeats of 250 ns unrestrained atomistic simulations were then performed.

For all atomistic MD simulations, the long-range electrostatics (< 1 nm) was modelled with the Particle Mesh Ewald (PME) method (Essmann et al., 1995). Temperature coupling was done with V-scale thermostat at 310 K. The Parrinello-Rahman barostat (Parrinello and Rahman, 1981) with a reference pressure of 1 bat and a compressibility of 4.5 × 10^−5^/bar was applied for pressure control. Covalent bonds are constrained to their equilibrium length by the LINCS algorithm (Hess et al., 1997). The integration steps of all simulations were set to 2 fs.

#### PMF Calculations

For PC2 system, protein truncation was done on the equilibrated PIP_2_ and PC systems. Equilibrations were performed with unbiased MD simulations for 100 ns. Position restraints with a force constant of 1000 kJ/mol/nm^2^ were used to prevent protein translation during equilibration.

For PC2, the reaction coordinates were defined as the distance between the COM of Ser505 and Ser591 of PC2 and the whole head group of PC, PS (N^∗^, PO^∗^ and GL^∗^ beads), PIP, PIP_2_ and PIP_3_ (RP^∗^, PO^∗^ and GL^∗^ beads). 38 umbrella sampling windows were evenly spaced on the reaction coordinates between -0.7 and 3 nm with a force constant of 500 kJ/mol/nm^2^. Position restraints (400 kJ/mol/nm^2^ in the *xy* plane) were applied to the backbone beads of Ser505 and Ser591 in each subunit to prevent rotation and translation of the protein. The reaction coordinates are approximately parallel to the X axis of the simulation box and a weak positional restraint of 100 kJ/mol/nm^2^ was applied to the corresponding pulling groups of the lipids to limit its movements along the Y axis.

The PLUMED2 package (Tribello et al., 2014) was used to define the reaction coordinates and apply biasing to pull the lipids. WHAM was used to unbias all umbrella sampling simulations. For systems of all PIPs, the dissociation study was done on one of the four bound lipid molecules. For PC system, the protein was placed in pure PC bilayers, and one PC molecule situated in one of the binding sites was chosen to be pulled away from the proteins. For the PS system, the pulled PC in the PC system was replaced with a PS molecule, and a similar pulling protocol was applied. Simulations were run for 3 μs per window for all PIP systems and for 2 μs for the PC and PS systems. To calculate the energies, the first 800 ns of each window was discarded for all PIP systems, and the first 500 ns for the PC and PS systems.

#### Free Energy Perturbation Calculations

CG FEP calculations were performed as described in (Corey et al., 2019), using 21 x 250 ns windows evenly spaced along the reaction coordinate, with 3 repeats run per system. Energies were computed on the final 225 ns of each window using multistate Bennett acceptance ratio, as implemented with alchemical analysis (Klimovich et al., 2015).

#### Simulation Visualisation and Analysis

Protein structures were visualised with VMD (Humphrey et al., 1996) or PyMOL (DeLano, 2002). Simulation trajectories were processed using GROMACS. Averaged lipid density maps were generated using the VMD Volmap plugin tool with three-dimensional grids every 0.1 nm.

#### Protein Expression and Purification

The human PKD2 gene which encodes the PC2 (polycystin-2, PC2 or TRPP1) protein was purchased from the Mammalian Gene Collection (MGC, 138466; IMAGE, 8327731, BC112263). A truncated stable construct (hPC2: Pro185 - Asp723) was used, with a C-terminal purification tag containing a TEV cleavage site, a His_10_ sequence, and a FLAG tag, was cloned into the expression vector pFB-CT10HF-LIC (available from The Addgene Nonprofit Plasmid Repository). DH10Bac competent cells were used for the production of baculovirus. Recombinant baculoviruses were used to infect *Spodoptera frugiperda* (Sf9) insect cells, grown in 250 mL suspension in Sf900II serum free, protein-free insect cell medium with L-glutamine (Thermo Fisher Scientific) at 27°C, when cell density reached ∼2 × 10^6^/ml for virus amplification at 27°C in 1 L shaker flasks. 1 L of Sf9 insect cells in Insect-XPRESS Protein-free Insect Cell Medium with L-glutamine (Lonza) in a 3 L flask was infected with 5 ml of the harvested P2 (second passage) viruses for 65 h at 27°C. Cells were harvested 65 h post-transduction by centrifugation for 15 min at 1500 g and 4°C.

Extraction buffer containing 50 mM HEPES, pH 7.5, 150 mM NaCl, 20 mM CaCl_2_, 5% glycerol and Roche protease-inhibitor cocktail was used to re-suspend the cell culture pellets to a volume of 50ml/L. Cells were lysed on ice with VCX 750 sonicator and 13mm probe (PRO Scientific Inc.) for 5 min, 3 sec on, 12 sec off and 35% amplitude. 1% (w/v) DDM was added to the cell lysis and incubated for 1 h at 4°C. Cell debris was removed by centrifugation for 1h at 35,000*g* and 4°C. To purify by immobilised metal affinity chromatography, the detergent-solubilised protein were batch bound to Co^2+^-charged Talon resin (Clontech) by gentle rotation at 4°C for 1 h. The resin was washed with 15 column volumes of extraction buffer supplemented with 0.01% DDM and 30 mM imidazole, pH 8.0 and, to exchange the detergent, with another 15 column volumes of the same buffer replacing DDM with 0.035% UDM. hPKD2:185-723 protein was eluted from Talon resin with extraction buffer supplemented with 0.035% UDM and 400 mM imidazole. The eluted protein was divided into two batches. One was further purified via size-exclusion chromatography (SEC) with a Superose 6 increase 10/300GL column (GE Healthcare) pre-equilibrated with SEC buffer (0.035% UDM, 20 mM HEPES, pH 7.5, 200 mM NaCl and 20 mM CaCl_2_) for PIP strip experiments. The other was buffer exchanged into SEC buffer using a PD-10 column (GE Healthcare The protein was then treated with 0.4 units of bovine kidney α-L-fucosidase (Sigma) at 18°C, overnight. The pH was adjusted to 7 before further enzymatic treatment at a ratio of 0.75:0.5:1 (w/w/w) TEV protease, PNGase F, PC2 for another 24 h at 18°C. Reverse His-tag purification was performed to clear out the His_6_-tagged TEV protease and uncleaved PC2. The protein was concentrated to 0.5 ml with a 100-kDa-cutoff concentrator (Vivaspin 20, Sartorius), and further purified by SEC as above.

#### PIP Strip Assay

PIP strip membranes (Thermo Fisher Scientific P23751) were blocked in 3% (w/v) fatty acid-free BSA (Sigma-Aldrich) in TBST (50 mM Tris-HCl, pH 7.5, 150 mM NaCl and 0.1% (v/v) Tween 20] for 1 h. The membranes were then incubated in the same solution with 0.5 μg/ml of detergent solubilised His_10_/FLAG-tagged PC2 overnight at 4°C with gentle agitation. The membranes were washed 3 times over 30 min in fatty acid-free BSA-TBST. One more 3h-incubation with 0.5 μg/ml PC2 and subsequent wash steps were performed at 4°C. The membranes were incubated for 1h with 1:2000 dilution of HRP conjugated anti-FLAG monoclonal antibody (Thermo Fisher Scientific MA1-91878-HRP) at room temperature. Finally, the membranes were washed 6 times over 1 h in TBST, and the protein that was bound to the membrane by virtue of its interaction with phospholipid was detected by enhanced chemiluminescence.

#### Cryo-EM Grid Preparation and Data Acquisition

18:0-20:4 PI(4,5)P_2_ and 18:1 PI(3,5)P_2_ lipid extract (Avanti) dissolved in chloroform was dried under an argon stream. 18:0-20:4 PI(4,5)P_2_ (∼ 0.5 mg/ml) and 18:1 PI(3,5)P_2_ (∼ 1 mg/ml) stock was prepared by resolubilising dried lipids in a buffer containing 20 mM HEPES (pH 7.5), and 150 mM NaCl via bath sonication for ∼1h. Purified PC2 was incubated with 18:0-20:4 PI(4,5)P_2_ at a molar ratio of 1:20 (PC2 tetramer: 18:0-20:4 PI(4,5)P_2_) and with 18:1 PI(3,5)P_2__2_ at a molar ratio of 1:40 (PC2 tetramer: 18:1 PI(3,5)P_2_) overnight at 4°C. The sample was cleared by centrifugation at 21,000g for 30 min. For cryo-EM, 3 μl of PC2 with PI(4,5)P_2_ sample at a protein concentration of ∼4 mg/ml or PC2 with PI(3,5)P_2_ sample at a protein concentration of ∼3.5 mg/ml was applied to glow-discharged Quantifoil 1.2/1.3 holey carbon 300 mesh copper grids. Grids were plunge frozen in liquid ethane using a Vitrobot Mark IV (FEI, Thermo Fisher Scientific) set to 5°C, 100% relative humidity, 3.5 s blotting time and -15 blotting force. Data for PC2 with PI(4,5)P_2_ were collected on a Titan Krios 300-kV transmission electron microscope equipped with a post-column Gatan image filter (GIF; 20eV slit width) operating in zero-loss mode and a Gatan K2 Summit direct electron detector camera at Central Oxford Structural Molecular Imaging Centre (COSMIC). Movies were captured for 0.4 s per frame over 8 s. The calibrated pixel size and dose rate were 0.822 Å/pix and ∼6.55 electrons/Å^2^/s, respectively (total dose 52.4e^-^/Å^2^). Images were collected in a defocus range between -1.0 and -3.0 μm under focus. Data for PC2 with PI(3,5)P_2_ were collected on a Titan Krios 300-kV transmission electron microscope at the Electron Bio-Imaging Centre (eBic, Diamond Light Source) equipped with a Gatan K2 Summit direct electron detector camera mounted behind a GIF and operated in zero-loss mode (0-20eV). Movies were captured for 0.2 s per frame over 7 s. The calibrated pixel size and dose rate were 0.816 Å/pix and ∼6.0 electrons/Å^2^/s, respectively (total dose 42e^-^/Å^2^). Images were collected in a defocus range between -1.0 and -3.1 μm under focus in 0.3 μm steps.

#### Image Processing

The beam-induced motion in the movies was corrected and frames were dose-weighted using MotionCor2 (Zheng et al., 2017). Aligned frames in each movie were averaged to produce a micrograph for further processing. The contrast transfer function (CTF) parameters were estimated using CTFFIND-4.1 (Rohou and Grigorieff, 2015). Micrographs with ice contamination or poor CTF cross correlation scores were discarded and the remaining micrographs were processed using RELION 3 (Kimanius et al., 2016). Particle picking was performed using Gautomatch (URL: http://www.mrc-lmb.cam.ac.uk/kzhang/).

For the dataset of PC2 with PI(4,5)P_2_, a set of 77,399 particles were picked from 1,597 micrographs and sorted into 2D classes. Representative 2D classes were used as templates for autopicking after low-pass filtering to 30 Å. A total of 147,001 particles were automatically picked. Three rounds of iterative 2D classification were performed in RELION to remove bad particles. A low-resolution reference model was generated *ab initio*. The initial model was used for the first round of 3D classification without symmetry imposed. A subset of 73,883 particles was used for subsequent 3D classification with C4 symmetry imposed. The data was then used for the first round of 3D ‘gold-standard’ refinement, which resulted in and initial reconstruction with a nominal unmasked resolution of 3.5 Å. Subsequent 3D classification was performed without further image alignments followed by iterative CTF refinement and Bayesian polishing. The final subset of particles was subjected to further auto-refinement in RELION, which converged at an unmasked resolution of 3.12 Å. RELION post-processing using unfiltered half maps and a soft-edged mask to exclude the region occupied by the detergent micelle yielded a final B-factor sharpened map (-84.56Å^2^) with a nominal resolution of 2.96 Å (FSC=0.143).

For the dataset of PC2 with PI(3,5)P_2_, a set of 323,538 particles were initially picked from 3,353 micrographs and classified into 2D classes. Representative well resolved class averages were used as templates for reference-based particle picking after low-pass filtering to 30 Å. A total of 224,402 particles were automatically picked using GAUTOMATCH. Three rounds of iterative 2D classification were performed in RELION to remove bad particles. A low-resolution reference model was generated *ab initio*. The initial model was used for the first round of 3D classification without symmetry imposed. One further round of 3D classification with C1 symmetry was performed on a set of 97,892 particles from classes with the best estimated resolutions. A subset of 91,844 particles was used for subsequent 3D classification with C4 symmetry imposed. The data was then used for the first round of 3D ‘gold-standard’ refinement, which resulted in and initial reconstruction at a resolution of 4.1 Å. A final set of 37,297 particles was selected from 3D classification performed without further image alignments. Iterative CTF refinement and Bayesian polishing were performed prior to a final auto-refinement procedure in RELION, which converged at an unmasked resolution of 3.66 Å. Subsequent post-processing, using a soft-edged mask that excluded the detergent micelle, produced a 3.4 Å resolution map. Reported resolutions were based on a FSC threshold of 0.143. Local resolutions across the whole map were estimated using RESMAP (Figure S14) (Kucukelbir et al., 2014).

#### Model Building

The previously published model of apo PC2 (PDB 5K47) was used as an initial model and fitted into the cryo-EM map of lipid-bound PC2. The model was manually adjusted in Coot (Emsley and Cowtan, 2004). In the original PDB 5K47 structure, the disulphide bond between Cys311 and Cys334 appeared to be reduced. In both PIP complexes, there is clear density showing that a disulphide bond is formed between these cysteines and has been built in both structures. One spherical density corresponding to the size of a dehydrated sodium or calcium ion is clearly present below the selectivity filter in each of the PIP complexes maps with the density deeper in the central pore in PI(3,5)P_2_ map compared to PI(4,5)P_2_ map. No such density is present in the equivalent location in map EMD-8354 (corresponding to PDB id 5T4D; (Shen et al., 2016)). This difference in density is likely to originate from the presence of calcium ions in our buffer which were not present in that for 5T4D/EMD-8354. Therefore, calcium ions were into the ion density in both of our structures. A series of *B*-factor sharpened maps were used to guide model building. Final models of both structures were globally refined and minimised in real space against the RELION3 C4 post-processed, automatically *B*-factor sharpened maps with NCS constraints, secondary structure and rotamer restraints imposed and no Ramachandran restraints applied using the *phenix.real_space_refine* module in PHENIX (Adams et al., 2010). The refinement protocol was validated by taking the final refined models, applying a random shift of up to 0.3Å to the atomic coordinates and then refining the resultant shifted model against the halfmap1. Model-to-map FSCs were calculated using *phenix.mtriage* against halfmap1 (FSCwork), halfmap2 (FSCfree) and the full map (FSCsum).

### Quantification and Statistical Analysis

Statistical analysis and software used can be found in the relevant sections of the methods and the figure legends.

### Data and Code Availability

This study did not generate new software. The simulation trajectory datasets supporting the current study have not been deposited as a public repository for MD simulation data does not yet exist. Coordinates of the models generated by this study (as representative frames from simulations revealing the interactions of PC2 with PIP_2_ and with cholesterol) are available from the corresponding author on request. Details of deposited coordinates and density are provided in the Key Resources Table. The accession numbers for the deposited coordinates reported in this paper are PDB: 6T9N, 6T9O.

## Supplemental Information

Document S1. Figures S1–S10 and Tables S1 and S2

Document S2. Article plus Supplemental Information
