# Supplementary material for: Lipid Interactions of a Ciliary Membrane TRP Channel: Simulation and Structural Studies of Polycystin-2
Source: Structure. 2020 Feb 4;28(2):169–184.e5. doi: 10.1016/j.str.2019.11.005 (PMC7001106; doi:10.1016/j.str.2019.11.005)
Supplement: Document S1. Figures S1–S10 and Tables S1 and S2 [file mmc1.pdf]

Structure, Volume *28*

Supplemental Information

Lipid Interactions of a Ciliary Membrane TRP Channel: Simulation and Structural Studies of Polycystin-2

Qinrui Wang, Robin A. Corey, George Hedger, Prafulla Aryal, Mariana Grieben, Chady Nasrallah, Agnese Baronina, Ashley C.W. Pike, Jiye Shi, Elisabeth P. Carpenter, and Mark S.P. Sansom

SUPPLEMENTAL FIGURES AND TABLES

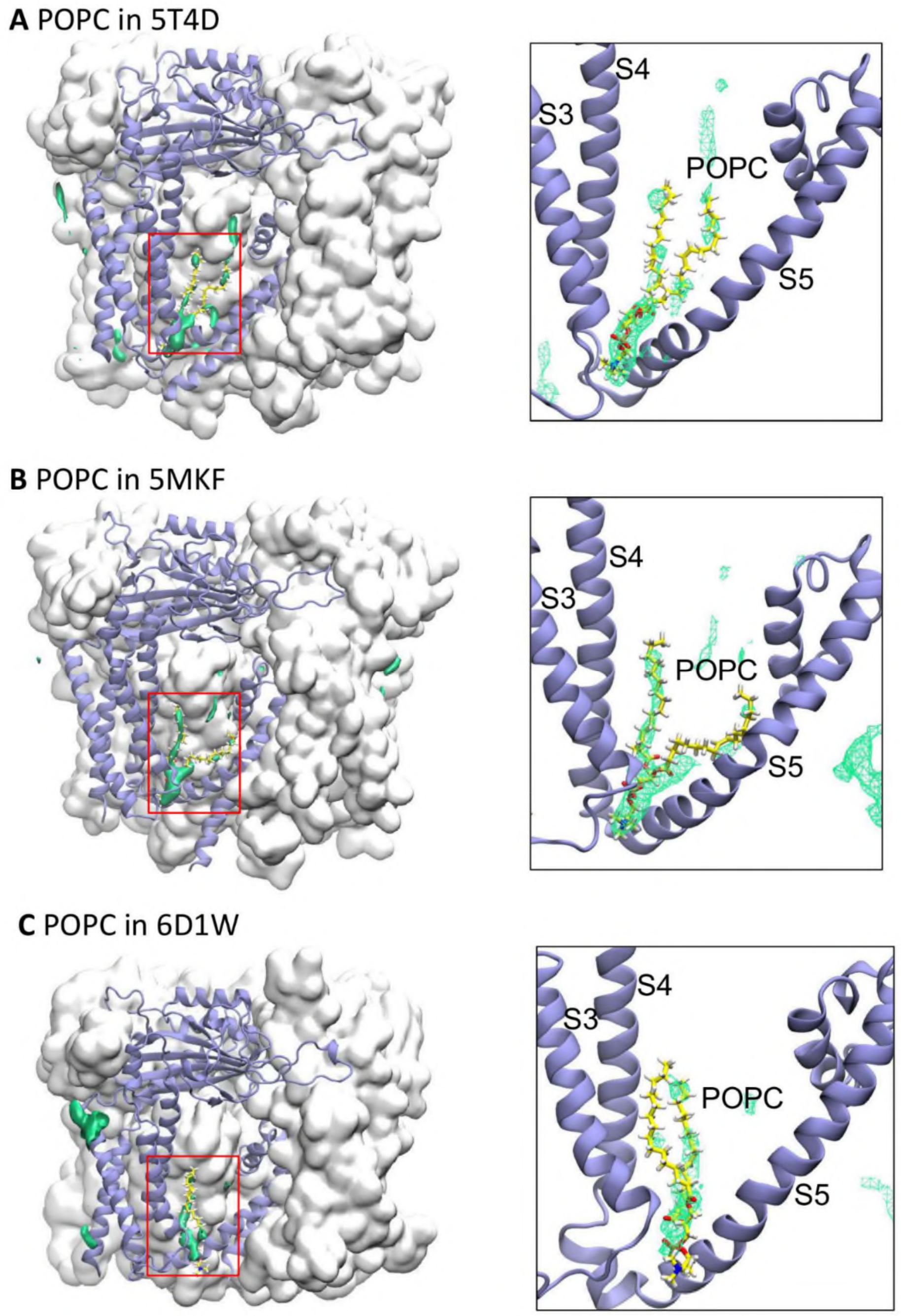

<u>Figure S1:</u> **Probability density of phosphatidylcholine in atomistic simulations. (Related to Figure 1.)**

Probability density of phosphatidylcholine in atomistic simulations based on three different PC2 structures: **A** 5T4D, **B** 5MKF, and **C** 6D1W. In each case in the left panel PC2 is shown as a grey surface, viewed perpendicular to the central pore axis, with one subunit depicted as a pale purple cartoon. Green isocontour surfaces represent a high probability of occurrence of phospholipid molecules. In the right panels, corresponding zoomed in views (red box) of the S3/S4/S5 pocket and the high phospholipid occurrence density are shown with a POPC molecule taken from a simulation snapshot.

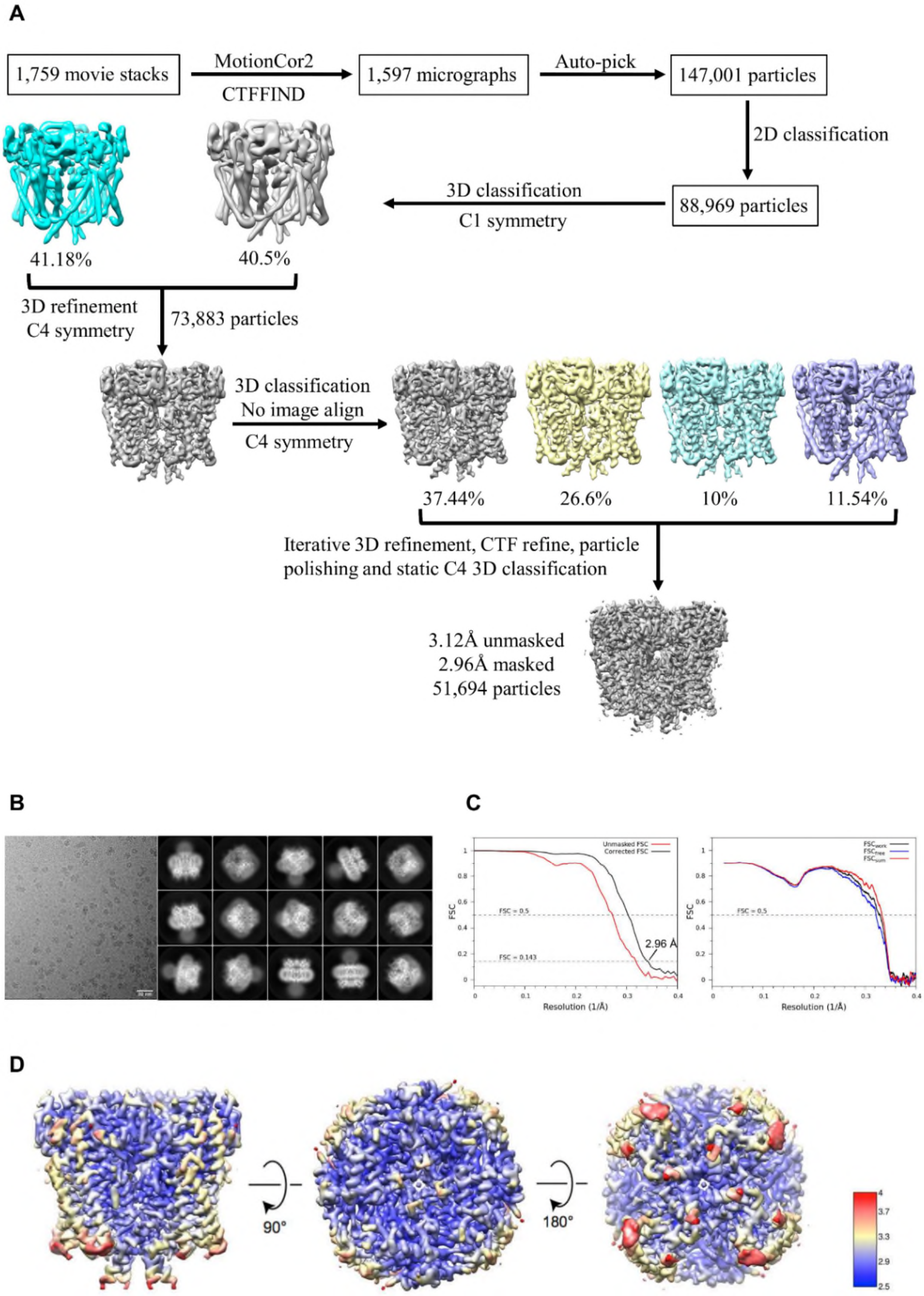

Figure S2: **PC2 structure with PI(4,5)P_2. (Related to Figure 2.)**

(**A)** Data processing for the PC2 dataset with PI(4,5)P_2 in detergent. Workflow of processing the images of PC2 with PI(4,5)P_2 to 3 Å resolution. (**B)** A representative raw micrograph and 2D classes of detergent-solubilized PC2 with PI(4,5)P_2. (**C**) Fourier shell coefficient (FSC) curves of the corrected (black) and unmasked (red) maps, obtained from RELION post-processing which have been corrected by phase randomisation. FSC of 0.143 and 0.5 are indicated with dotted lines. FSC curves for cross validation comparing the model to half maps 1 (black) and 2 (blue), and to summed map (red dotted). (**D**) Local resolution of the final density map in three different orientations on a scale of 2.5 Å (blue) to 4 Å (red).

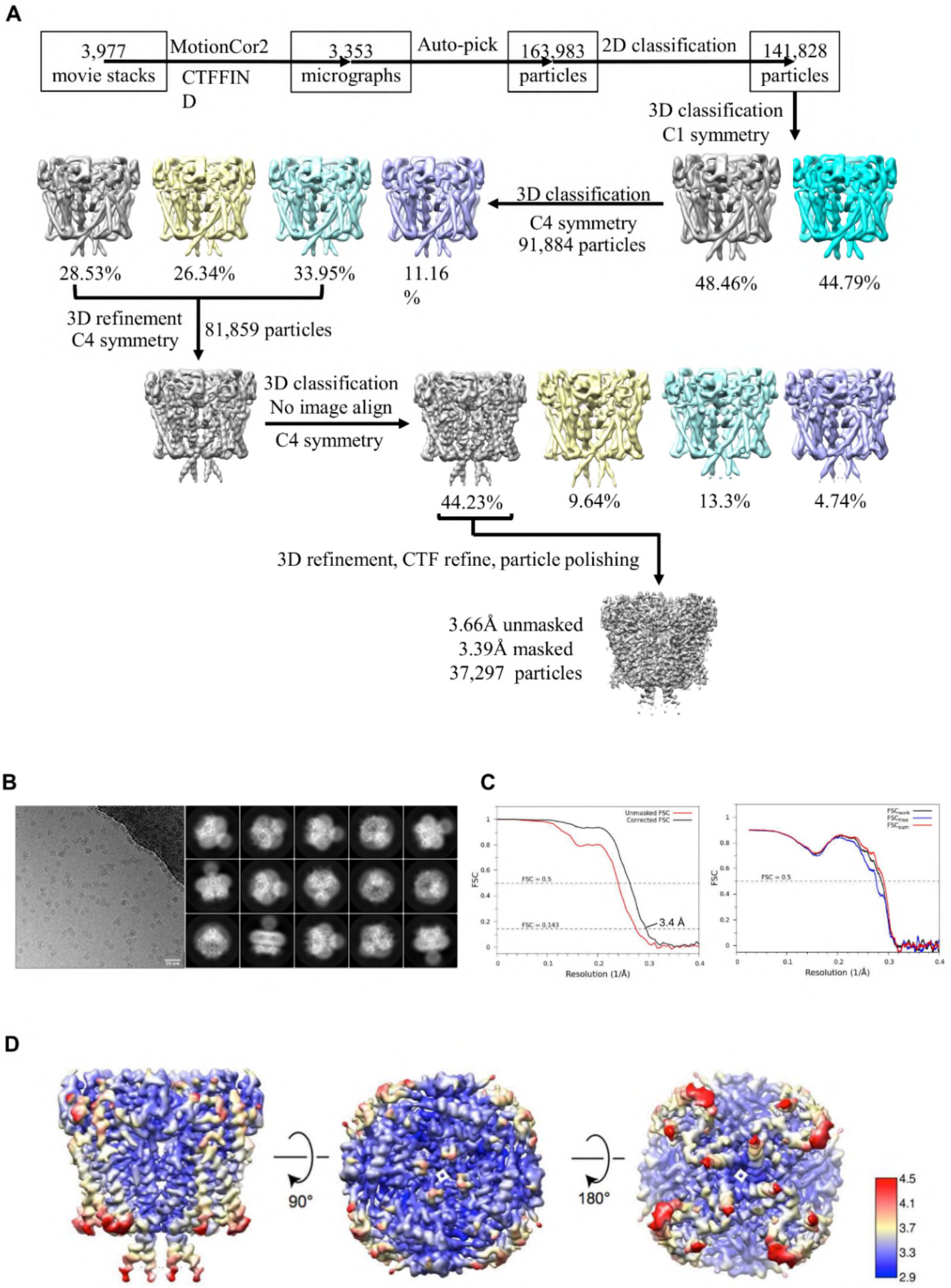

Figure S3: **PC2 structure with PI(3,5)P$_2$. (Related to Figure 2.)**

(**A**) Data processing for the PC2 dataset with PI(3,5)P$_2$ in detergent. Workflow of processing the images of PC2 with PI(3,5)P$_2$ to 3.4 Å resolution. (**B)** A representative raw micrograph and 2D classes of detergent-solubilized PC2 with PI(3,5)P$_2$. (**B**) Fourier shell coefficient (FSC) curves of the corrected (black) and unmasked (red) maps, obtained from RELION post-processing which have been corrected by phase randomisation. FSC of 0.143 and 0.5 are indicated with dotted lines. FSC curves for cross validation comparing the model to half maps 1 (black) and 2 (blue), and to summed map (red dotted). (**D**) Local resolution of the final density map in three different orientations on a scale of 2.9 Å (blue) to 4.5 Å (red).

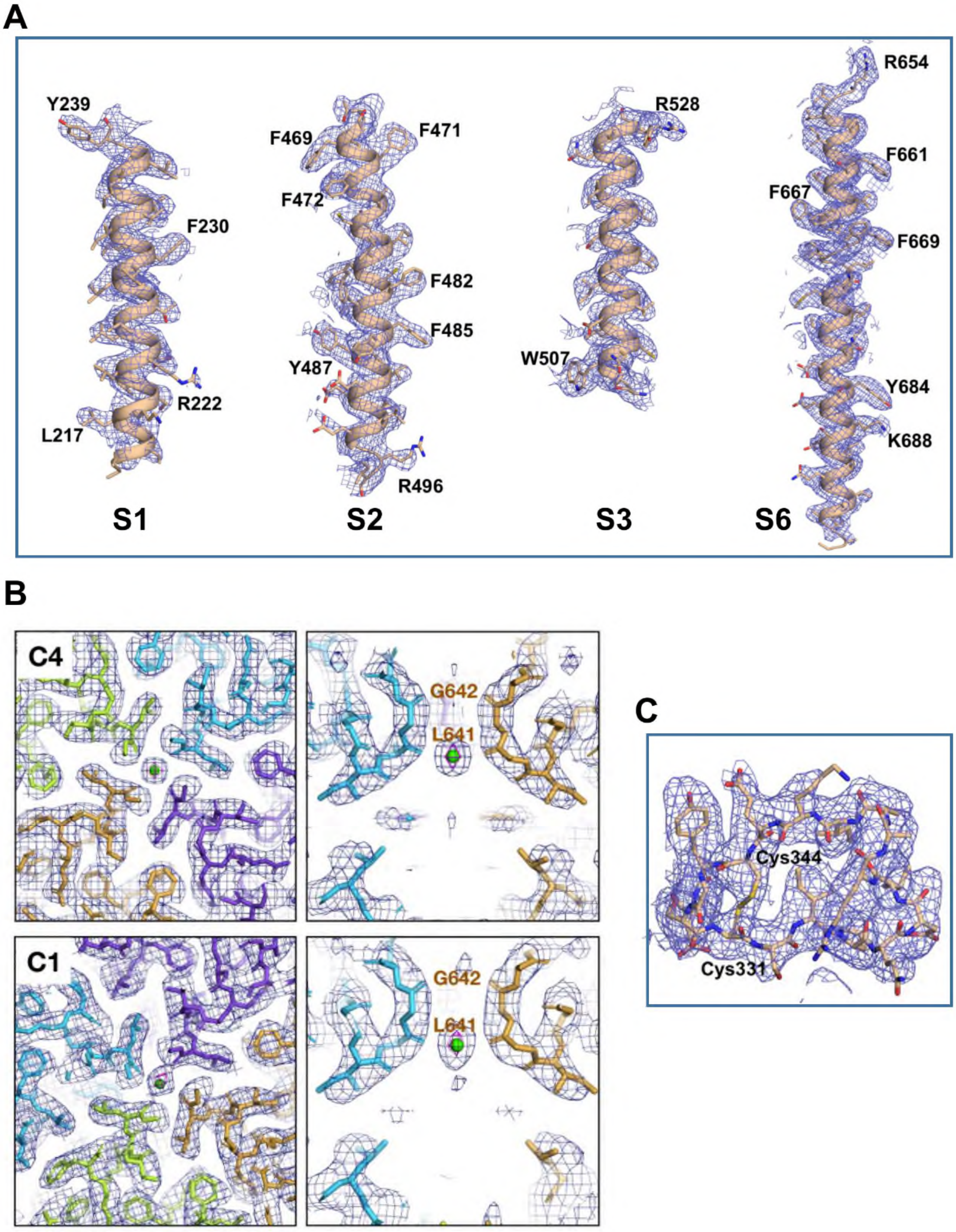

<u>Figure S4:</u> **Model and density for the 3 Å resolution structure. (Related to Figure 3.)**
(**A**) Density (blue) and model (ribbon plus sidechains) are shown for the S1, S2, S3 and S6 transmembrane helices. Density is shown filtered to 2.96 Å at 2σ. See main figure 3 for the corresponding figures for S4 and S5. (**B**) Density and structure in the region of the bound cation in the ion-conduction pathway from the C4 map and C1 maps derived from the 3 Å resolution structure (PC2/PI(4,5)P_2) dataset. The modelled cation is shown in green. Protein density (dark blue) is shown at 3σ. The putative ion density is contoured at 3σ (dark blue) and at 8σ (magenta). (**C**) Density and model for residues from Gly329 to Tyr345 showing the disulphide bond between Cys331 and Cys344. Density is shown filtered to 2.96 Å at 2σ.

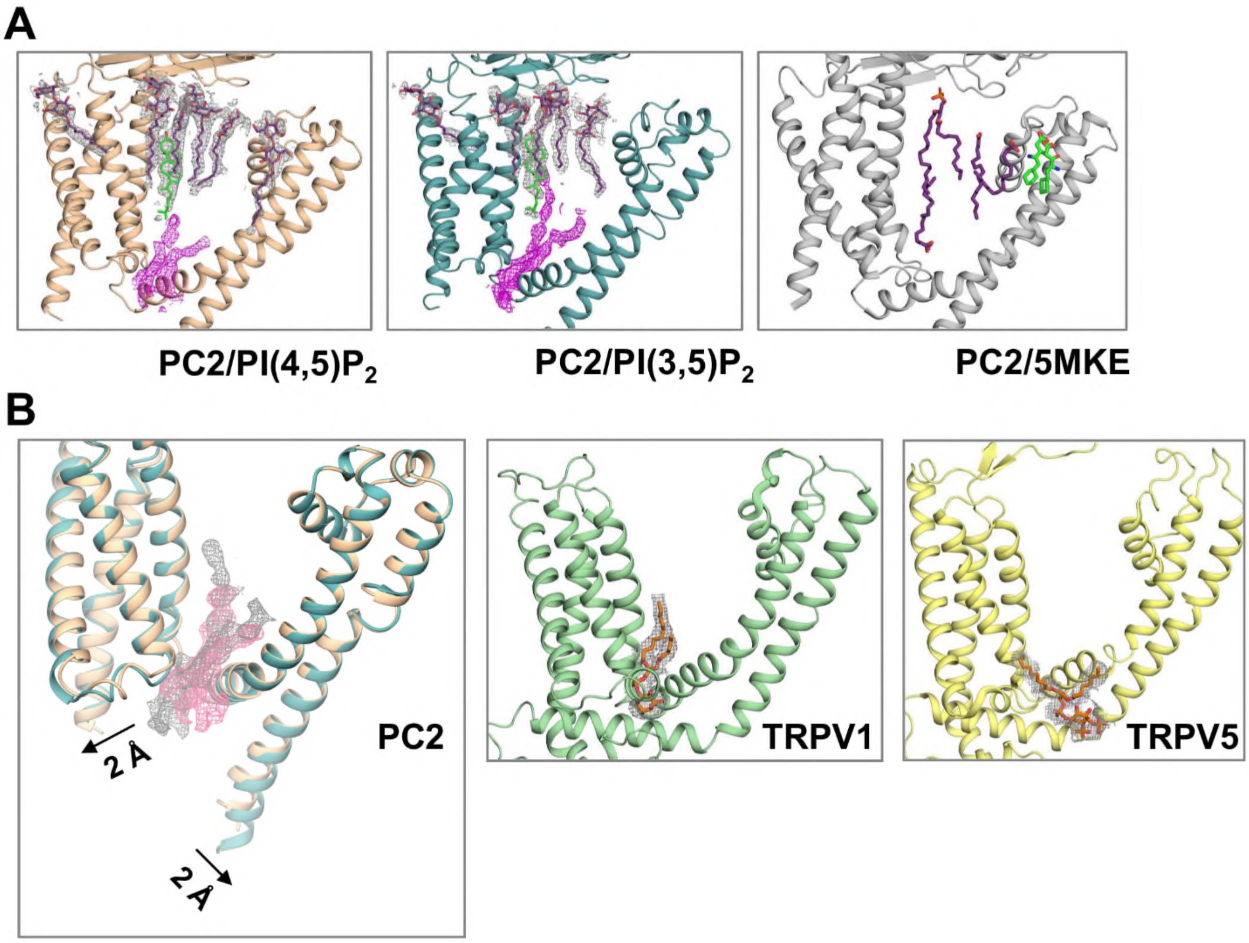

Figure S5: **Lipids and detergents in the new PC2 structures compared with those in other structures. (Related to Figures 2, 3, 7 and 8.)**

(**A)** Detergents and lipid bound in the 3.0 Å resolution PC2/PI(4,5)P_2 structure, in the 3.4 Å resolution PC2/PI(3,5)P_2 structure, and lipids bound in the previously published PC2 structure (PDB ID: 5MKE). (**B)** Lipid density comparison between PC2, TRPV1 and TRPV5. For PC2, an expanded view is shown around the potential lipid binding site between S3, S4 and S5 with density from the 3.0 Å (pink) and the 3.4 Å (grey) resolution maps. The PC2/PI(4,5)P_2 structure is shown in light orange cartoon format and the PC2/PI(3,5)P_2 structure is shown in light teal. For TRPV1 density and structure are shown for a phosphatidylinositol lipid molecule (PDB ID: 5IRZ; EMD-8118), for TRPV5 for a PI(4,5)P_2 molecule (PDB ID: 6DMU; EMD-7966). For PC2 we observed a small outward movement (~2 Å) of the S2-S3 linker region and comparable inward movement of S6 helix in the PI(3,5)P_2 structure compared to the PI(4,5)P_2 structure as indicated by the arrows. The main chain RMSDs between the overall PC2 structures are: PC2/PI(4,5)P_2 vs. PC2/PI(3,5)P_2 = 0.6 Å; PC2/PI(4,5)P_2 vs. 5T4D = 0.6 Å; and PC2/PI(3,5)P_2 vs. 5T4D = 0.6 Å.

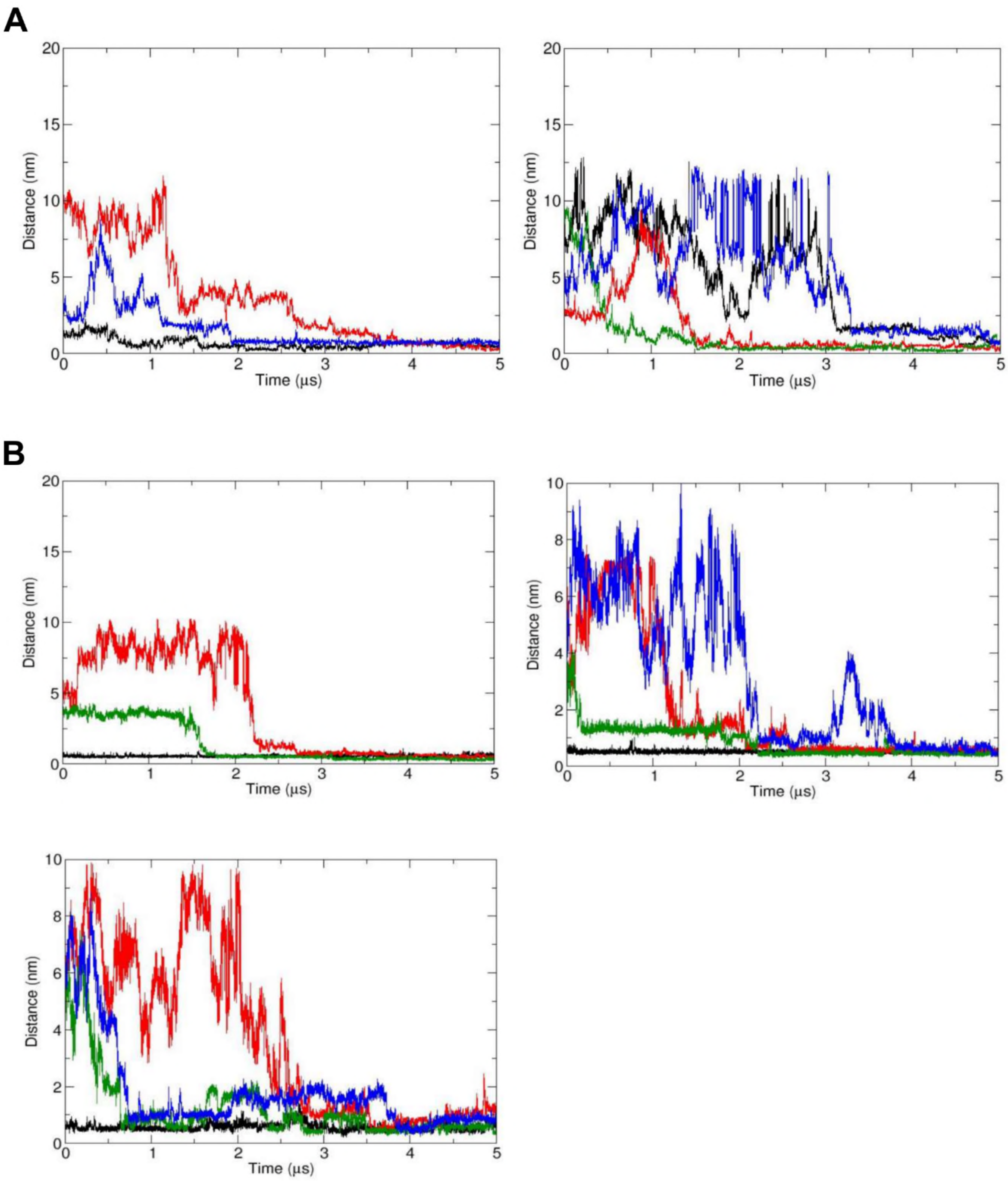

__Figure S6:__ **PIP_2 binding in simulations of PC2. (Related to Figure 4.)**

(**A**) PIP_2 binding in two repeats of a coarse-grained (CG) simulations of PC2 (PDB id: 5K47) in an *in vivo* mimetic mixed lipid bilayer (also see Fig. 2A of the main paper for the third simulation of this triplicate). The distances from their binding site on PC2 of PIP_2 molecules are shown as functions of time. Distances are measured from the centre of mass of the headgroup of each PIP_2 molecule to the centre of mass of residues S505 and S591 of the site to which that lipid molecule eventually binds. The different coloured lines correspond to the different PIP_2 molecules. (**B**) PIP_2 binding in three repeats of CG simulations of a gain-of-function Phe604Pro mutant of PC2 (PDB id: 6D1W) in an *in vivo* mimetic mixed lipid bilayer.

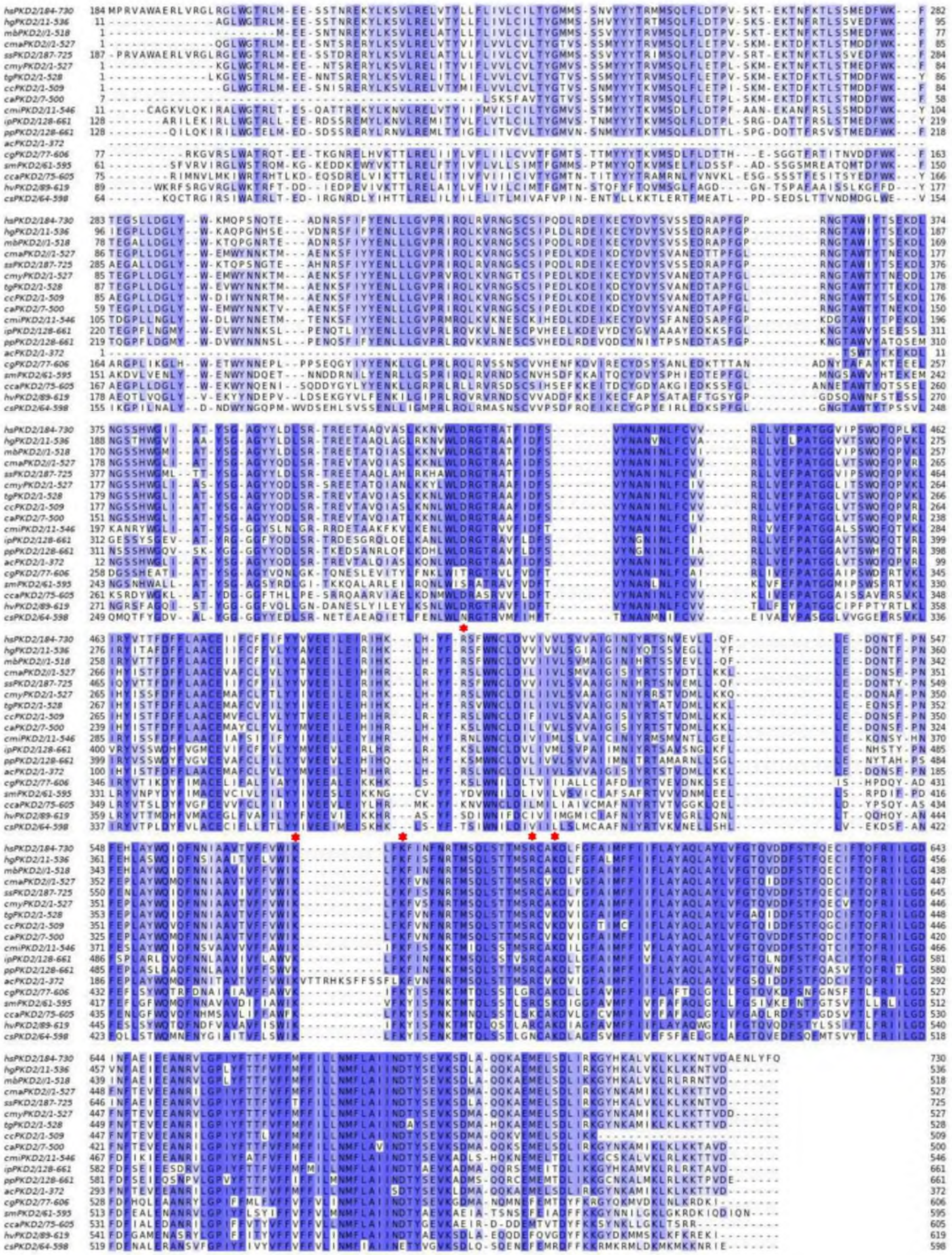

Figure S7: **Sequence alignment for PC2 from 18 different species. (Related to Figure 4.)**
The sequence of human PC2 (M184-Q730) (hsPKD2) is aligned with the corresponding sequences from *H. glaber* (hg), *M. brandtii* (mb), *C. macqueenii* (cma), *S. scrofa* (ss), *C. mydas* (cmy), *T. guttatus* (tg), *C. canorus* (cc), *C. anna* (ca), *C. milii* (cmi), *I. punctatus* (ip), *P. prolifica* (pp), *A. chloris* (ac), *C. gigas* (cg), *S. mimosarum* (sm), *C. candelabrum* (cca), *H. vulgaris* (hv), *C. sinensis* (cs). Residues Arg504, Lys572, Lys575, Arg592 and Lys595, identified to coordinate the PIP_2 headgroup in human PC2, are highlighted by red stars.

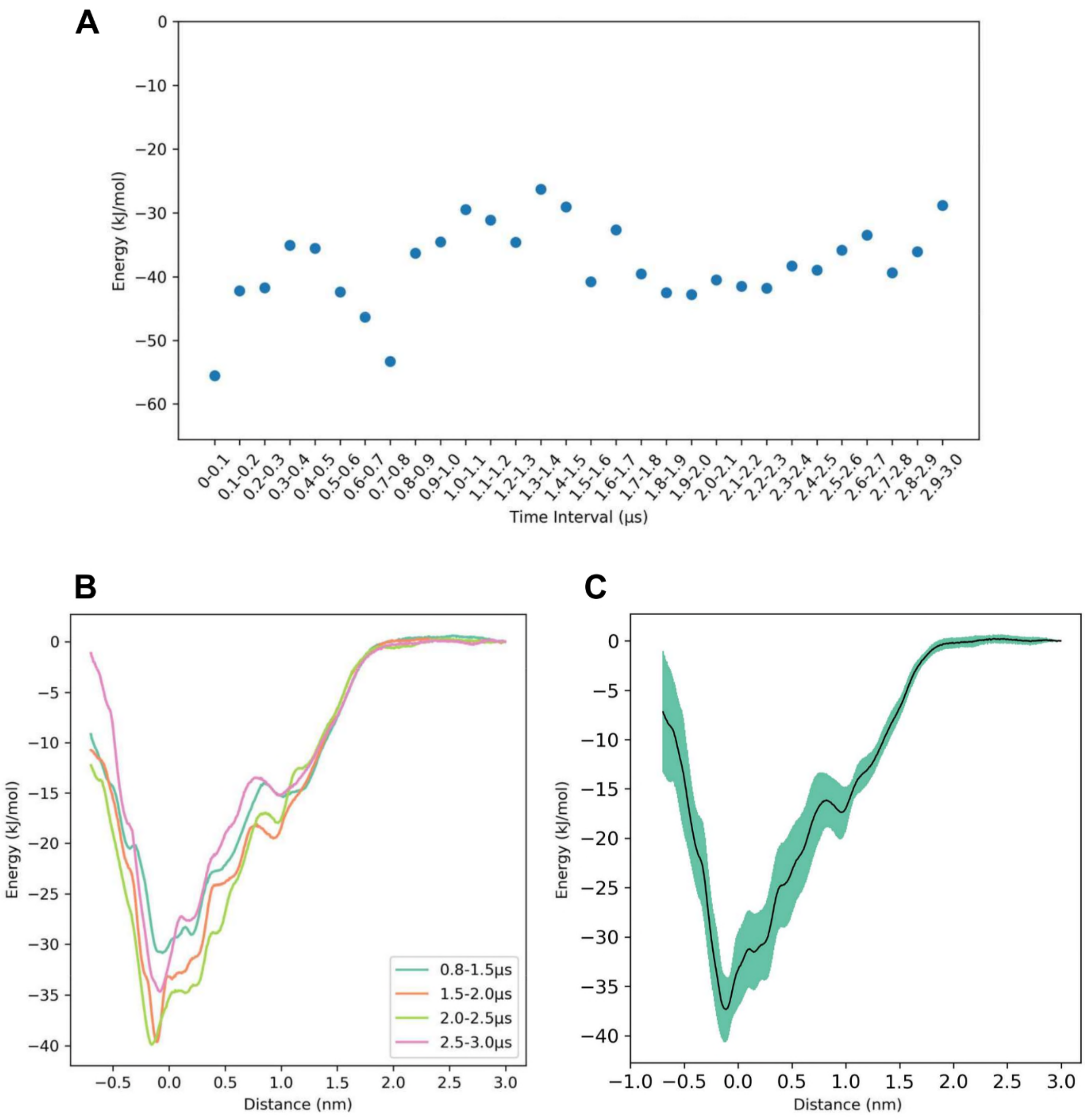

Figure S8: **Convergence of PMF calculations of PIP$_2$ binding to PC2. (Related to Figure 5.)** (**A)** Convergence of a PMF calculation for PIP$_2$ binding was assessed by calculating the binding free energy (measured as the energy well depth) derived from non-overlapping 100 ns segments of the simulations. (**B)** PMF profiles calculated with non-overlapping 500 to 700 ns time segments are shown. The first 800 ns of each window were excluded from the PMF calculation. (**C**) Average (black) PMF and standard deviation (green) calculated from PMFs for non-overlapping 200 ns time segments from 1.6 to 3.0 μs.

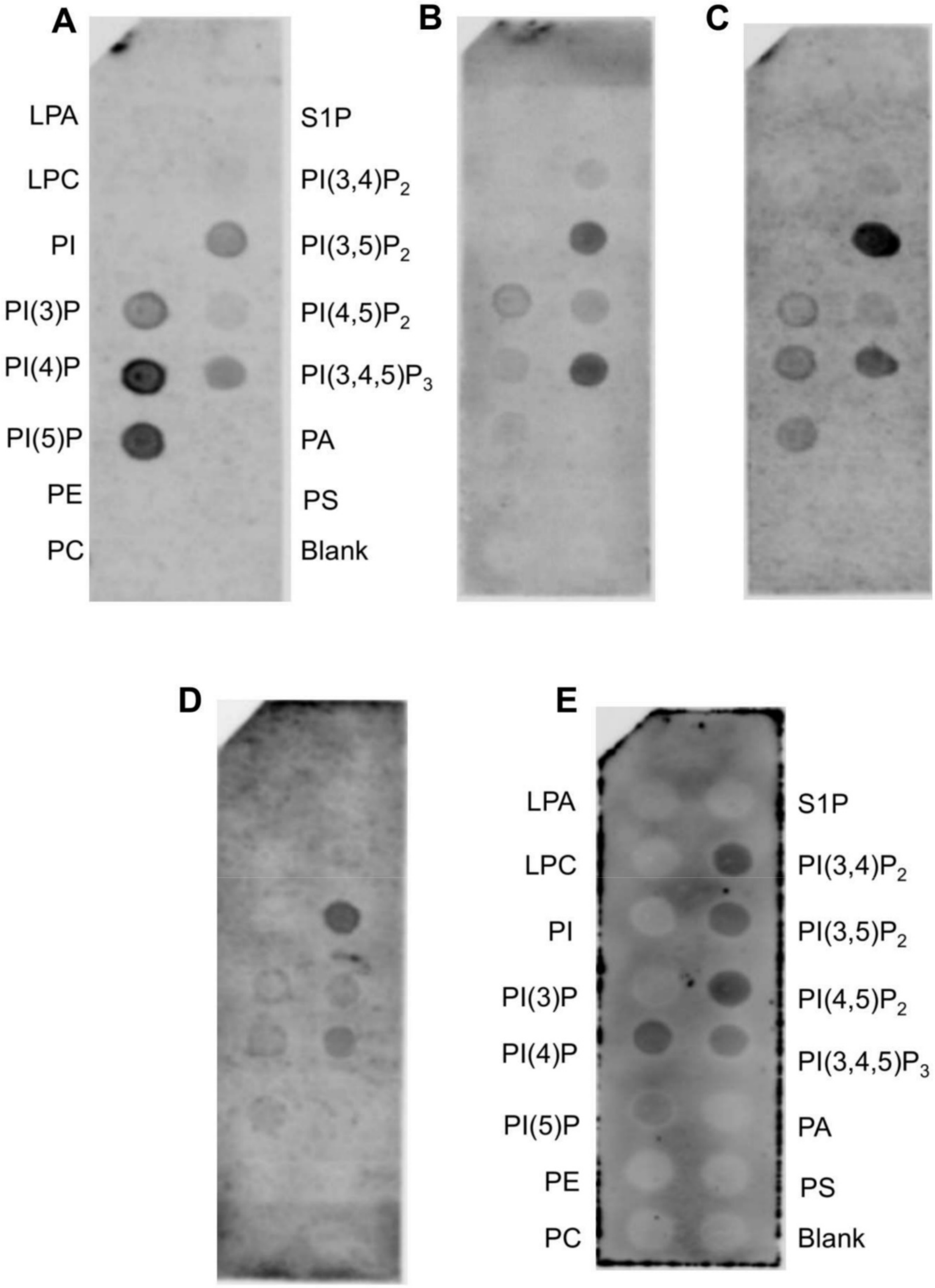

Figure S9: **Biological repeats of PC binding to PIP strips. (Related to Figure 5.)**
PC2 expressed in different batches of either GNT- HEK (**A**) or Sf9 (**B - E**) cells were used to repeat the PIP strip experiments. Although binding intensities vary between the different repeats, the bound lipid species are broadly consistent.

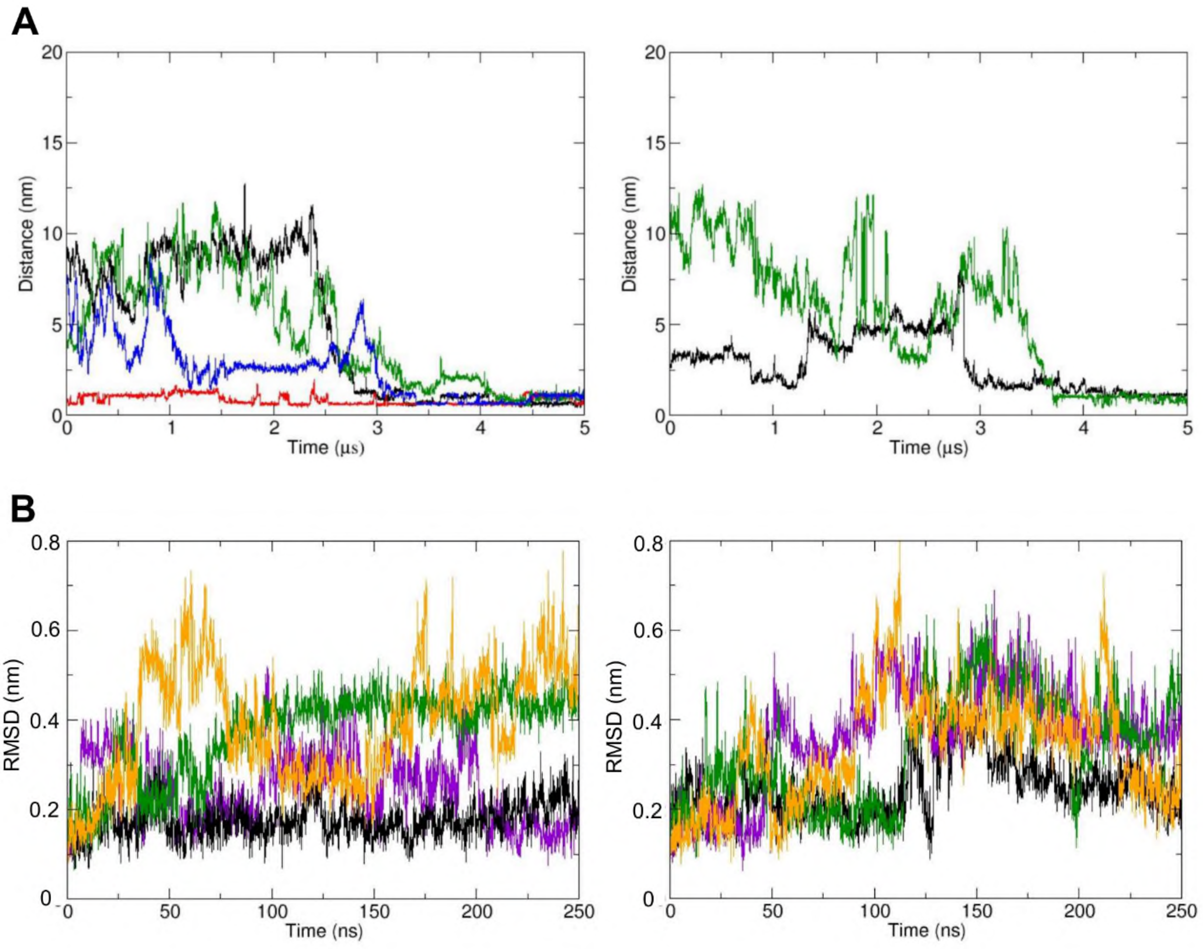

<u>Figure S10:</u> **Cholesterol binding in simulations of PC2. (Related to Figures 8 and 9.)**

(**A**) Cholesterol binding in two repeats of CG simulations of PC2 (PDB id: 5K47) in an *in vivo* mimetic mixed lipid bilayer. Distances from the binding site as function of time for cholesterol molecules are shown. The distance shown is from the centre of mass of each cholesterol molecule to the centre of mass of Gln557 and Asn560 at the site to which that lipid molecule eventually binds. Different coloured lines correspond to the different cholesterol molecules. (**B**) Two repeats of atomistic simulations of cholesterol-bound PC2, with the cholesterol molecule initially built into the cryoEM density. RMSD vs. time for the four cholesterol molecules bound to PC2 during 250 ns atomistic MD simulations are shown. Different coloured lines correspond to the different cholesterol molecules.

Table S1: Summary of Simulations. (Related to Figures 1, 4, 5, 6, 8, and 9.)

Channel	PDB ID	Bilayer composition	Replicates x duration (μs)
Atomistic			
PC2	5K47	PC	3 x 0.2
PC2	5T4D	PC	3 x 0.25
PC2	5MKF	PC	3 x 0.25
PC2/F604P	6D1W	PC	3 x 0.25
PC2 + PIP_2	5K47	PC	3 x 0.25
PC2 + Chol§	3 Å PI(4,5)P_2 dataset	PC	3 x 0.25
Coarse Grained			
PC2	5K47	mixed lipid bilayer*	3 x 5
PC2/F604P	6D1W	mixed lipid bilayer	3 x 5
PKD2L1	6DU8	mixed lipid bilayer	3 x 5
TRPA1	3J9P	mixed lipid bilayer	2 x 5
TRPC3	6CUD	mixed lipid bilayer	3 x 5
TRPM2	6C07	mixed lipid bilayer	3 x 5
TPRM4	6BQV	mixed lipid bilayer	3 x 5
TRPML1	5WJ5	mixed lipid bilayer	3 x 5
TRPML1	5WJ9	mixed lipid bilayer	3 x 5
TRPML3	6AYF	mixed lipid bilayer	3 x 5
TRPV1	3J5P	mixed lipid bilayer	2 x 10
TRPV2	5AN8	mixed lipid bilayer	3 x 5
TRPV5	6B5V	mixed lipid bilayer	3 x 5
TRPV6	6B08	mixed lipid bilayer	3 x 5

§To be deposited
*The mixed lipid bilayer contained PC:PE:SM:GM3:CHOL = 40:10:15:10:25 in the outer leaflet and PC:PE:PS:PIP_2:CHOL = 10:40:15:10:25 in the inner.

Table S2: Cryo-EM data, refinement and model statistics. (Related to STAR Methods)

**Cryo-EM data**	PI(4,5)P_2	PI(3,5)P_2
**Data Collection:**		
Voltage (kV)	300	300
Defocus range (µm)	-3 to -1 in 0.25 increments	-3.1 to -1 in 0.3 increments
Pixel size (Å)	0.822	0.816
Electron dose (e^-/Å^2)	52.4	42.05
Dose rate (e^-/Å^2/s)	6.55	6.0
Number of micrographs (used)	1,597	3,353
Particles (initial)[1]	98,113	163,983
Particles (final, %)	51,694 (52.7)	37,297 (22.7)
**Reconstruction:**		
Symmetry	C4	C4
Resolution (unmasked, Å)[2]	3.12	3.66
Resolution (masked, Å)[2]	**2.96**	**3.39**
Map sharpening *B*-factor (Å^2)	-84.56	-108.53
**Model composition:**		
Protein atoms	15,668	15,612
Other	1,249	869
**Refinement:**		
Resolution (Å)	**3**	**3.4**
Map sharpening factor (Å^2)	-84.56	-108.53
Fourier Shell Correlation (FSC)[3]	0.8485	0.8309
**Rms deviations:**		
Bonds (Å)	0.011	0.009
Angles (°)	1.002	0.875
**Molprobity validation:**		
Clashscore, all atoms	**3.72**	**3.63**
Molprobity score	1.43	1.61
Ramachandran Plot (% favoured)	96.01	92.66
Ramachandran Plot (% allowed)	3.57	6.71
Ramachandran Plot (% outliers)	0.42	0.63

[1]Particles after one cycle of 2D classification to remove non-particles
[2]Based on FSC 0.143 threshold
[3] CC_mask from *phenix_real_space_refine*
